# EGFR inhibition leads to enhanced desmosome assembly and cardiomyocyte cohesion via ROCK activation

**DOI:** 10.1172/jci.insight.163763

**Published:** 2023-03-22

**Authors:** Maria Shoykhet, Orsela Dervishi, Philipp Menauer, Matthias Hiermaier, Sina Moztarzadeh, Colin Osterloh, Ralf J. Ludwig, Tatjana Williams, Brenda Gerull, Stefan Kääb, Sebastian Clauss, Dominik Schüttler, Jens Waschke, Sunil Yeruva

**Affiliations:** 1 Chair of Vegetative Anatomy, Institute of Anatomy, Faculty of Medicine, Ludwig Maximilian University (LMU), Munich, Germany.; 2 Lübeck Institute of Experimental Dermatology and Center for Research on Inflammation of the Skin, University of Lübeck, Lübeck, Germany.; 3 Comprehensive Heart Failure Center and Department of Medicine I, University Hospital Würzburg, Würzburg, Germany.; 4 Medizinische Klinik und Poliklinik I, LMU Hospital, LMU, Munich, Germany.; 5 DZHK (German Centre for Cardiovascular Research), Partner Site Munich, Munich Heart Alliance (MHA), Munich, Germany.; 6 Interfaculty Center for Endocrine and Cardiovascular Disease Network Modeling and Clinical Transfer (ICONLMU), LMU Munich, Munich, Germany.; 7 Institute of Surgical Research at the Walter-Brendel-Centre of Experimental Medicine, LMU Hospital, LMU, Munich, Germany.

**Keywords:** Cardiology, Cell Biology, Cardiovascular disease, Cell migration/adhesion

## Abstract

Arrhythmogenic cardiomyopathy (AC) is a familial heart disease partly caused by impaired desmosome turnover. Thus, stabilization of desmosome integrity may provide new treatment options. Desmosomes, apart from cellular cohesion, provide the structural framework of a signaling hub. Here, we investigated the role of the epidermal growth factor receptor (EGFR) in cardiomyocyte cohesion. We inhibited EGFR under physiological and pathophysiological conditions using the murine plakoglobin-KO AC model, in which EGFR was upregulated. EGFR inhibition enhanced cardiomyocyte cohesion. Immunoprecipitation showed an interaction of EGFR and desmoglein 2 (DSG2). Immunostaining and atomic force microscopy (AFM) revealed enhanced DSG2 localization and binding at cell borders upon EGFR inhibition. Enhanced area composita length and desmosome assembly were observed upon EGFR inhibition, confirmed by enhanced DSG2 and desmoplakin (DP) recruitment to cell borders. PamGene Kinase assay performed in HL-1 cardiomyocytes treated with erlotinib, an EGFR inhibitor, revealed upregulation of Rho-associated protein kinase (ROCK). Erlotinib-mediated desmosome assembly and cardiomyocyte cohesion were abolished upon ROCK inhibition. Thus, inhibiting EGFR and, thereby, stabilizing desmosome integrity via ROCK might provide treatment options for AC.

## Graphical abstract

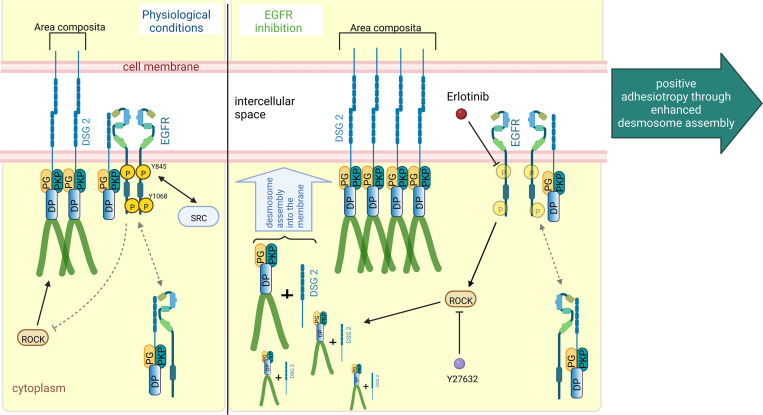

## Introduction

The epidermal growth factor receptor (EGFR) is a ubiquitously expressed transmembrane receptor tyrosine kinase. Dysregulation of EGFR signaling often results in pathologies, such as different kinds of cancer, as well as inflammatory skin and bowel disease or other skin, inflammatory, or renal disorders (1–7). Today, EGFR inhibitors, either small inhibitory molecules (e.g., erlotinib, gefitinib, or lapatinib) or inhibitory antibodies (e.g., cetuximab or panitumumab) are approved for the treatment of non–small cell lung cancer, pancreatic cancer, breast cancer, colorectal carcinoma, and squamous cell carcinoma of head and neck (8, 9). EGFR can be transactivated through G protein–coupled receptors via second messengers, several protein kinases, or matrix metalloproteases (10, 11). In addition, EGFR signaling can be activated, enhanced, and mediated by the proto-oncogene tyrosine-protein kinase Src (SRC) (12). Conversely, SRC can be activated by EGFR (13). In the heart, EGFR was found to play a crucial role in cell metabolism, proliferation, and cell survival as well as hypertrophy and remodeling (14). EGFR inhibition can lead to heart failure in the healthy heart, and a total KO of the receptor can lead to severe hypertrophy and hypotension (15–17). However, under pathological conditions, EGFR inhibition can be cardioprotective, as EGFR has been shown to be upregulated in diabetes (18). Interestingly, EGFR inhibition protected vasculature and heart from diabetes-induced damages (19–23). Furthermore, recent studies indicate that EGFR inhibition has a protective effect on cardiomyocytes under hypoxic conditions — i.e., after myocardial infarction (24). It seems that, like in other tissues, EGFR signaling is tightly controlled in the heart, and due to its double role and the multitude of its downstream effectors, any alterations in EGFR signaling can have severe effects on heart function (25, 26).

Arrhythmogenic cardiomyopathy (AC) is a disease most commonly caused by mutations in genes coding for desmosomal proteins, such as desmoglein 2 (DSG2), plakoglobin (PG), desmoplakin (DP), desmocollin 2 (DSC2), N-Cadherin (N-CAD), and plakophilin 2 (PKP2) (27, 28). Therefore, AC is considered as a hereditary disease of the desmosome. AC-causing mutations often lead to alterations in desmosomal contacts between cells, thereby weakening cellular cohesion. Several pathways regulating cardiomyocyte cohesion have been identified (29–33). Furthermore, there is increasing evidence that desmosomes not only provide cellular cohesion, but also act as signaling hubs (34, 35). Thus, cardiac desmosomes are part of a complex signaling network, which can either influence and/or are influenced by cellular signaling processes. Recent advancement in research suggests that similar molecular mechanisms are present in AC and pemphigus, an autoimmune skin disease, as well as inflammatory bowel diseases (33, 34, 36–38).

In the context of pemphigus, EGFR inhibition led to increased cellular cohesion in keratinocytes (39), whereas in squamous carcinoma cell lines, EGFR inhibition increased protein levels of DSG2 and DSC2 (40). However, EGFR inhibition by erlotinib in intestinal epithelial cells caused a decrease in cellular cohesion (41), revealing that the role of EGFR can vary between different cells or tissues.

EGFR seems to play a major role in the heart function. Furthermore, links between EGFR and DSG2 or EGFR and other desmosomal proteins have been established. Thus, in this study, we investigated whether inhibition of the EGFR signaling pathway can be used as a therapeutic option for AC, via regulating cardiomyocyte cohesion, using the *Jup*^–/–^ mouse model (*Jup* is the gene coding for PG), which we characterized previously (31, 33, 42). To this end, we used the well-known and clinically approved first-generation EGFR inhibitor erlotinib and an EGFR signaling molecule SRC inhibitor, PP2. Erlotinib was used to directly inhibit EGFR, and PP2 was used to indirectly inhibit EGFR by inhibiting the EGFR signaling pathway, since SRC acts both up- and downstream of EGFR. This approach was used as a proof of concept that EGFR inhibition, either using drug or a signaling inhibitor, rescues impaired cardiomyocyte cohesion.

## Results

### Inhibition of EGFR or SRC lead to positive adhesiotropy in HL-1 cardiomyocytes and murine cardiac slice cultures.

**Figure 1:**
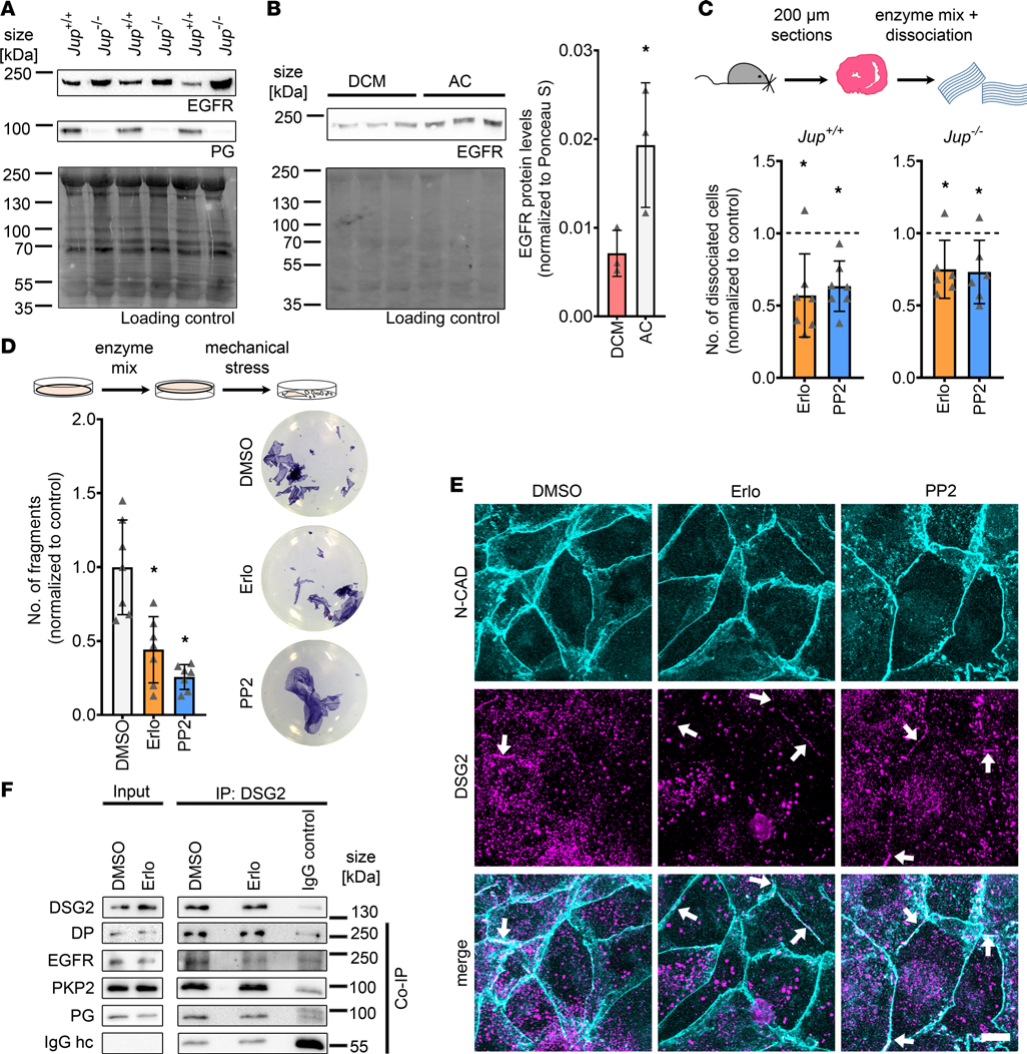
EGFR or SRC inhibition induces positive adhesiotropy in cardiomyocytes. (**A**) Representative Western blot showing protein expression of EGFR and PG in *Jup*^+/+^ and *Jup*^–/–^ mice, NoStain Protein Labeling Reagent was used as loading control. *n* = 6. (**B**) Western blot showing expression of EGFR in patients with AC and dilated cardiomyopathy (DCM). Ponceau S staining was used as a loading control. *P ≤ 0.05 unpaired Student’s *t* test, *n* = 3 patients per group. (**C**) Dispase-based dissociation assay in murine cardiac slice cultures obtained from *Jup*^+/+^ and *Jup*^–/–^ mice upon inhibition of EGFR or SRC. Consecutive slices were taken for controls and treatments, and treatments were normalized to the respective control slice to minimize variability due to differences in cardiac slice size. *P ≤ 0.05, 1-way ANOVA with Holm-Sidak correction, *n* = 7 for *Jup*^+/+^ mice and *n* = 6 for *Jup*^–/–^ mice. (**D**) Dispase-based dissociation assay in HL-1 cardiomyocytes upon inhibition of EGFR or SRC by erlotinib or PP2, respectively, with representative pictures of the wells. *P ≤ 0.05, 1-way ANOVA with Holm-Sidak correction, *n* = 7 independent experiments. (**E**) Maximum projections of immunostainings in HL-1 cardiomyocytes for N-CAD and DSG2 showing an increase of DSG2 at the cell borders after erlotinib or PP2 treatments. Z-scans spanning the whole cell volume; *z* steps = 0.25 μm. White arrows indicate areas of increased DSG2 localization at the cell membrane. Scale bar: 10 μm. (**F**) Representative Western blots for immunoprecipitation of DSG2, coimmunoprecipitation of DP, EGFR, PKP2, and PG. IgG heavy chain (IgG hc) served as loading control for immunoprecipitated samples. *n* = 3 independent experiments.

In keratinocytes and intestinal epithelial cells, as well as in cancer cells, it is known that EGFR can modulate the desmosomal structure, assembly, and signaling and vice versa (40, 41, 43–45). We used the cardiomyocyte-specific *Jup*^–/–^ mouse, representing a pathogenic AC model, which shows fibrotic replacement of myocardial tissue as well as arrhythmias (31, 33, 42). In this model, we found increased EGFR protein expression compared with WT (*Jup*^+/+^) mice (Figure 1A and Supplemental Figure 1A; supplemental material available online with this article; https://doi.org/10.1172/jci.insight.163763DS1). We also observed increased EGFR protein levels in heart lysates from a cardiomyocyte-specific *Pkp2*-KO mouse (Supplemental Figure 1B). To validate our findings in patients with AC, we assessed EGFR protein levels in lysates obtained from the atrial appendage of hearts from patients with AC and observed increased EGFR protein levels compared with dilated cardiomyopathy (DCM) hearts (Figure 1B).

We next investigated the role of EGFR in cardiomyocyte cohesion. To this end, we inhibited EGFR directly using erlotinib or inhibited SRC using PP2, thereby inhibiting the EGFR pathway, and performed dispase-based dissociation assays with murine cardiac slice cultures and HL-1 cardiomyocytes. Therefore, treated murine cardiac slices or HL-1 cell monolayers were subjected to mechanical stress, in order to challenge cell-to-cell contacts, upon which single cardiomyocytes (for murine cardiac slices) or fragments of the monolayer (for HL-1 cardiomyocytes) resulted.

We show that both EGFR or SRC inhibition enhanced cardiomyocyte cohesion, which we term as positive adhesiotropy, in murine cardiac slice cultures and HL-1 cardiomyocytes (Figure 1, C and D). Inhibition of EGFR or SRC was confirmed by assessing the phosphorylation levels of their downstream targets: ERK and EGFR (Supplemental Figure 2, A–C). We also performed immunostaining of N-CAD and DSG2 in HL-1 cardiomyocytes and documented an increase of DSG2 at the cell borders after EGFR or SRC inhibition, compared with controls (Figures 1E and Supplemental Figure 2, D–F). Next, to assess whether EGFR is in a complex with desmosomal proteins, we immunoprecipitated DSG2 from HL-1 lysates and performed immunoblots for EGFR, PG, DP, and PKP2. Indeed, EGFR was found in complex with DSG2 in addition to PG, DP, and PKP2 (Figure 1F). Nevertheless, treatment with erlotinib did not alter the composition of this complex.

These experiments showed that inhibition of either EGFR or SRC leads to positive adhesiotropy in HL-1 cardiomyocytes and murine cardiac slice cultures, paralleled by enhanced DSG2 localization along cell junctions.

### Inhibition of EGFR increases the DSG2 binding event frequency at cell borders.

**Figure 2:**
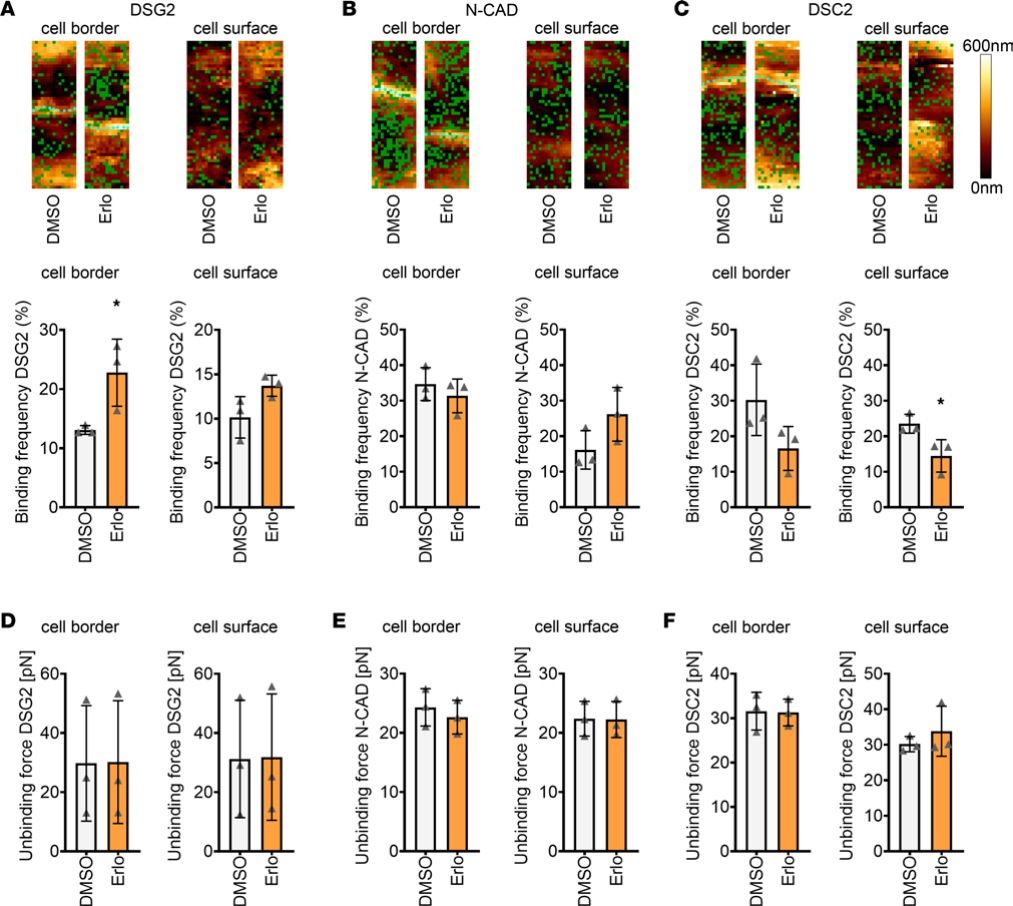
EGFR inhibition leads to increased DSG2 binding frequency at cell borders. (**A**–**C**) Binding frequency and topography during atomic force microscopy (AFM) measurements on HL-1 cardiomyocytes before and after erlotinib treatment at or close to cell borders and cell surfaces with tips coated with DSG2 (**A**), N-CAD (**B**), and DSC2 (**C**). Measurements were first performed in 90-minute DMSO-treated samples; then, medium was changed, erlotinib was added, and experiments were performed after 90 minutes. Green dots in topography images indicate binding events; cyan dots indicate cell borders. Topography images are 1.5 × 5 μm. In the graphs, per data point, 1,500 curves were analyzed across 2 areas (1.5 × 5 μm). *P ≤ 0.05. Statistical significance was calculated between DMSO versus erlotinib. Unpaired Student’s *t* test, *n* = 3 independent experiments. (**D**–**F**) Unbinding forces measured during AFM measurements on HL-1 cardiomyocytes at cell borders and cell surfaces with tips coated with DSG2 (**D**), N-CAD (**E**), and DSC2 (**F**). Per data point, 1,500 curves were analyzed across 2 areas (1.5 × 5 μm). *P ≤ 0.05. Statistical significance was calculated between DMSO versus erlotinib. Unpaired Student’s *t* test, *n* = 3 independent experiments.

Since we observed an increase in DSG2 localization at the membrane after EGFR or SRC inhibition, we assessed the role of DSG2 in strengthening cellular cohesion upon EGFR inhibition in HL-1 cardiomyocytes by atomic force microscopy (AFM) measurements. Here, a cantilever with a protein-coated tip approached the cells, where the protein at the tip could eventually interact with proteins at the cell surface. We measured the binding frequency of DSG2-, DSC2-, or N-CAD–coated tips as well as the unbinding forces at cell borders and cell surfaces. We observed an increase in binding frequency at cell borders for DSG2-coated tips only (Figure 2A). In the case of N-CAD–coated tips, no change in binding frequency was observed (Figure 2B). However, in the case of DSC2-coated tips, reduced binding frequency at the cell surface, but not the cell border, was found (Figure 2C). On the other hand, no changes in unbinding forces for any of the proteins analyzed were observed (Figure 2, D–F). These data indicate that enhanced binding frequency of DSG2 correlates with the strengthening of intercellular adhesion in HL-1 cardiomyocytes upon EGFR inhibition.

### Apart from DSG2, positive adhesiotropy induced by EGFR or SRC inhibition is also dependent on DP.

**Figure 3:**
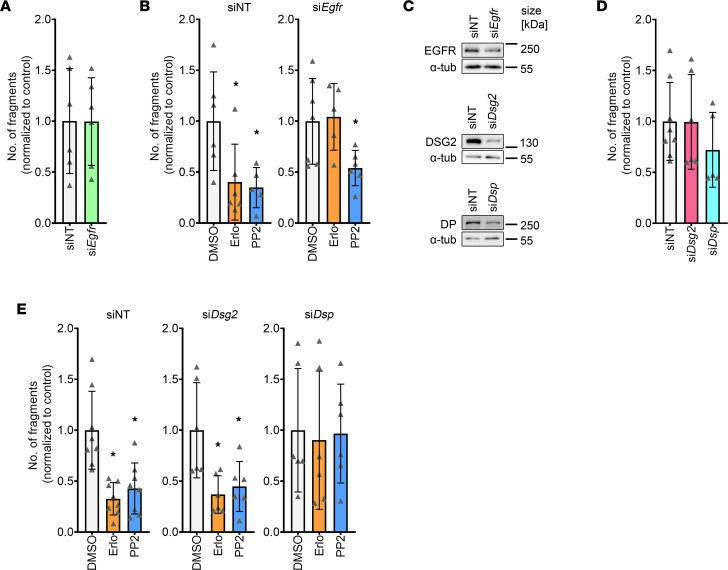
Positive adhesiotropy induced by EGFR or SRC inhibition is dependent on DP. (**A**) Dispase-based dissociation assay in HL-1 cardiomyocytes after siRNA-mediated knockdown of *Egfr* and nontarget (NT) control knockdown. Unpaired Student’s *t* test, *n* = 6 independent experiments. (**B**) Dispase-based dissociation assay in HL-1 cardiomyocytes after siRNA-mediated knockdown of EGFR and treatments with erlotinib or PP2. *P ≤ 0.05, 1-way ANOVA with Holm-Sidak correction, *n* = 6. (**C**) Representative Western blot confirmation of siRNA-mediated knockdown efficiency for *Egfr*, *Dsp*, and *Dsg2* knockdowns; α-tubulin served as loading control. *n* = 6 independent experiments. (**D**) Dispase-based dissociation assay in HL-1 cardiomyocytes after siRNA-mediated knockdown of *Dsg2* or *Dsp*. Unpaired Student’s *t* test. (**E**) Dispase-based dissociation assay in HL-1 cardiomyocytes after siRNA-mediated knockdown of *Dsg2* or *Dsp* and treatments with erlotinib or PP2. *P ≤ 0.05, 1-way ANOVA with Holm-Sidak correction, *n* = 6–8 independent experiments.

To further understand the mechanism of erlotinib- or PP2-induced positive adhesiotropy, we performed siRNA-mediated knockdown in HL-1 cardiomyocytes. Firstly, we knocked down *Egfr*, which did not alter cellular cohesion (Figure 3A and Supplemental Figure 3, A and B). We then knocked down *Egfr* and treated the cells with erlotinib or PP2. Upon *Egfr* knockdown, erlotinib-induced positive adhesiotropy was abolished, confirming that the effect observed after erlotinib treatment was mediated through EGFR. Interestingly, SRC inhibition was still effective after *Egfr* knockdown, indicating that the PP2 effect is either independent of EGFR or that SRC is downstream of EGFR (Figure 3B and Supplemental Figure 3A). Next, we knocked down *Dsp* (the gene coding for DP) or *Dsg2* (Figure 3C and Supplemental Figure 3, C and D). Knocking down *Dsp* or *Dsg2* did not alter cellular cohesion (Figure 3D and Supplemental Figure 3, E and F), as observed before (33, 42). Both erlotinib or PP2 enhanced cellular cohesion after *Dsg2* knockdown. EGFR or SRC inhibition–mediated positive adhesiotropy was abrogated upon *Dsp* knockdown, indicating the crucial role of DP in erlotinib or PP2-mediated positive adhesiotropy (Figure 3E and Supplemental Figure 3F). These data highlight the role of DP, along with DSG2, in positive adhesiotropy induced by EGFR or SRC inhibition.

### EGFR or SRC inhibition leads to increased area composita length in HL-1 cardiomyocytes.

**Figure 4:**
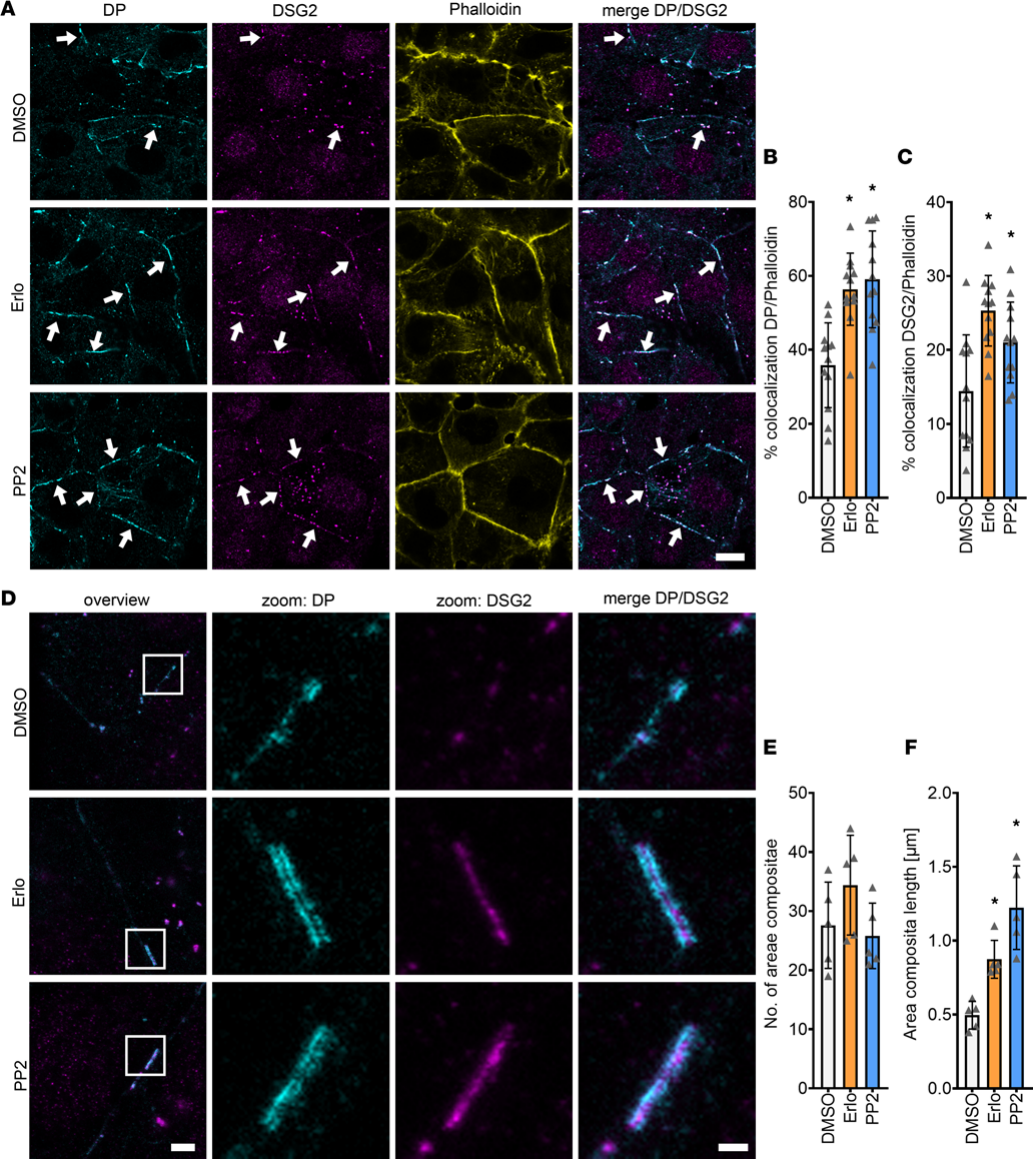
Increased DP and DSG2 recruitment to the cell membranes leads to longer areae compositae upon EGFR or SRC inhibition. (**A**) Immunostaining of DP and DSG2 in HL-1 cardiomyocytes with phalloidin as membrane marker. Scale bar: 10 μm. White arrows represent an increase in DSG2 and DP localization to the membrane compared with DMSO. (**B**) Quantification of colocalization of DP and phalloidin. *P ≤ 0.05, 1-way ANOVA with Holm-Sidak correction. (**C**) Quantification of colocalization of DSG2 and phalloidin. *P ≤ 0.05, 1-way ANOVA with Holm-Sidak correction. Each data point represents 1 area. *n* = 6 independent experiments. (**D**) STED images of HL-1 cardiomyocytes stained for DP and DSG2. Scale bar: 2 μm (for overview images), 500 nm (for zoomed images). (**E**) Number of areae compositae over 6 areas (15 × 15 μm) from *n* = 5 independent experiments. 1-way ANOVA with Holm-Sidak correction. (**F**) Quantification of area composita length. *P ≤ 0.05, 1-way ANOVA with Holm-Sidak correction, *n* = 5 independent experiments.

**Figure 5:**
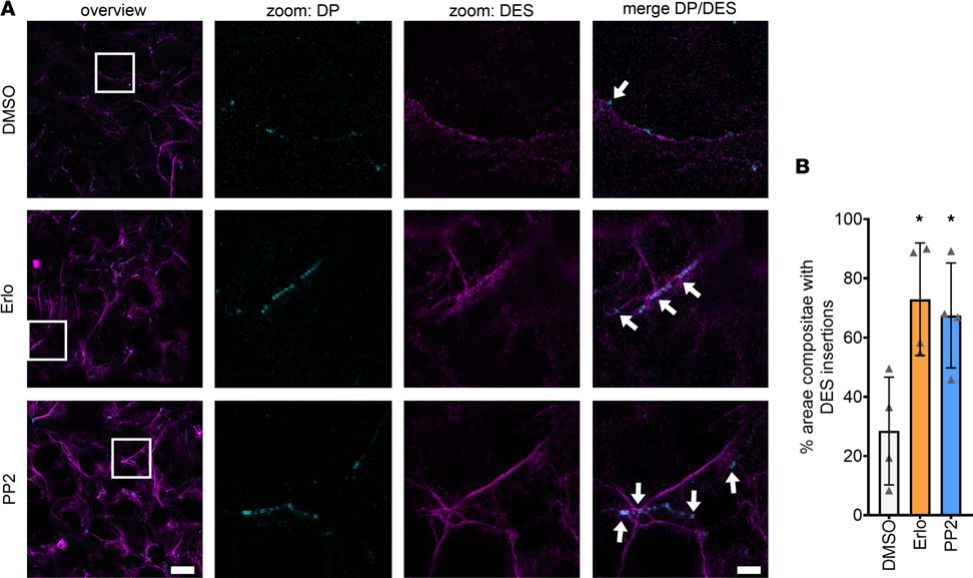
Increased DES insertion into DP upon erlotinib or PP2 treatments. (**A**) Representative STED images of HL-1 cardiomyocytes stained for DP and DES. (**B**) Bar graphs represent percentage of desmosomes with DES insertions. Scale bar: 10 μm (for overview images), 2 μm (for zoomed images). *P ≤ 0.05, 1-way ANOVA with Holm-Sidak correction, *n* = 4 independent experiments. White arrows indicate DES insertions into areae compositae.

Since both DSG2 and DP seem to be involved in the positive adhesiotropy induced by EGFR or SRC inhibition, we next performed immunostaining of these proteins together with phalloidin to stain the cortical actin cytoskeleton. Confocal microscopy images revealed an increase in the localization of DP and DSG2 at cell borders after inhibition of EGFR or SRC (Figure 4, A–C). At the same time, DSG2 and DP staining looked enlarged in erlotinib- and PP2-treated cells compared with vehicle controls, indicating changes at the area composita, that were shown to be present in intercellular junctions of HL-1 cells (30). Therefore, we performed stimulated emission depletion (STED) imaging for DSG2 and DP, and it showed that area composita number remained unchanged, whereas the area composita length increased after EGFR or SRC inhibition (Figure 4, D–F). Apart from that, we observed an increase in desmin (DES) insertion into area compositae upon treatment with erlotinib or PP2 (Figure 5, A and B) without changes in DES protein levels (Supplemental Figure 4, A and B).

### Enhanced DP and DSG2 staining in murine cardiac slices after EGFR or SRC inhibition.

**Figure 6:**
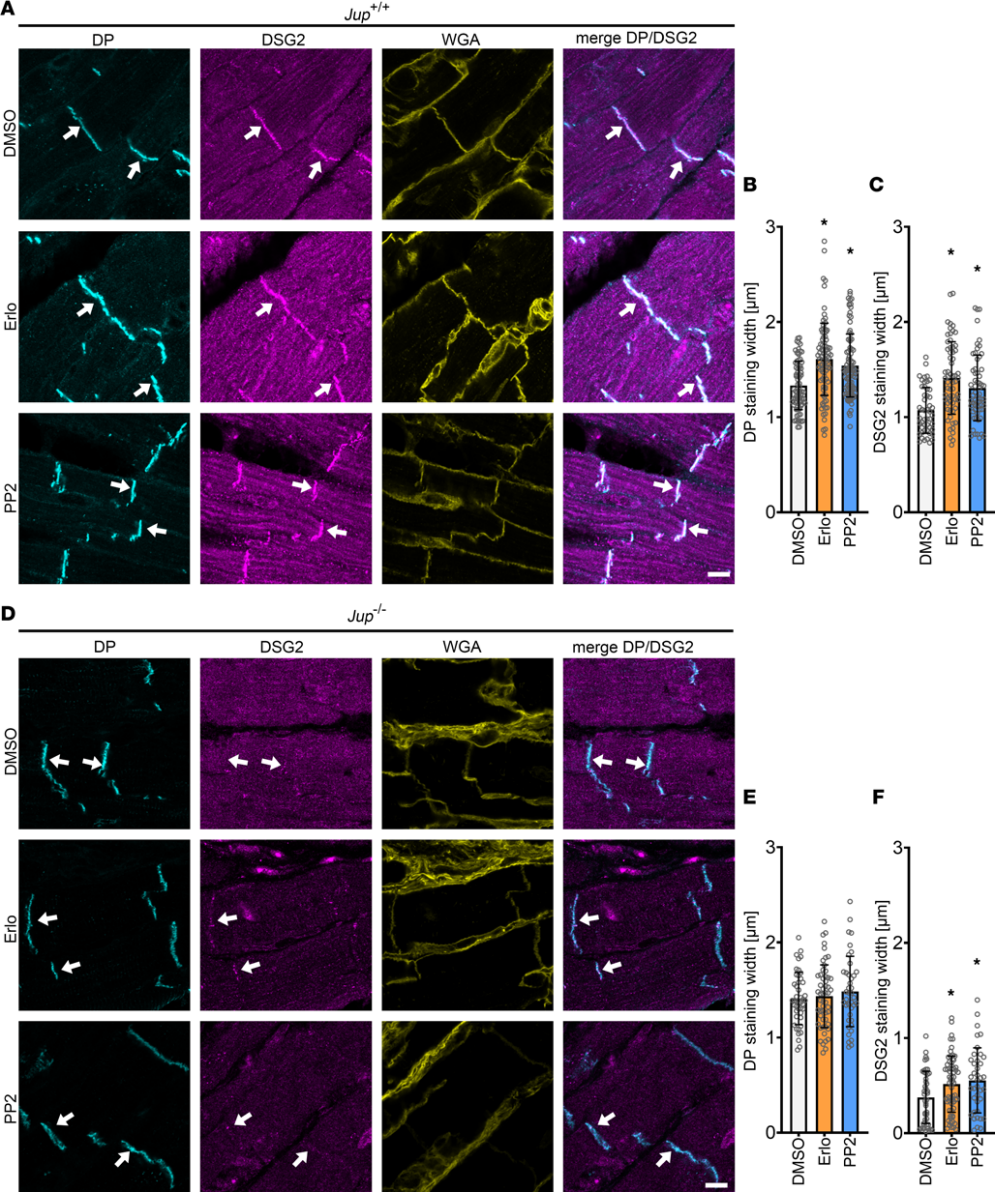
EGFR or SRC inhibition leads to increased recruitment of DP and DSG2 into the ICDs in *Jup*^+/+^ and *Jup*^–/–^ mice. (**A**) Immunostaining of DP and DSG2 in *Jup*^+/+^ murine cardiac slices with WGA as membrane marker. Scale bar: 8 μm. (**B**) DP staining width. (**C**) DSG2 staining width. *P ≤ 0.05, 1-way ANOVA with Holm-Sidak correction. Each data point represents 1 ICD. *n* = 4 mice. (**D**) Immunostainings of *Jup*^–/–^ murine cardiac slices for DP and DSG2 with WGA as membrane marker. Scale bar: 8 μm. (**E**) DP staining width. (**F**) DSG2 staining width. *P ≤ 0.05, 1-way ANOVA with Holm-Sidak correction. Each data point represents 1 ICD. *n* = 4 mice. White arrows represent an increase in staining width in DSG2 or DP staining compared with DMSO.

To confirm the findings from HL-1 cardiomyocytes in a mouse model, we performed immunostaining of murine cardiac slice cultures, both in *Jup^+/+^* and in *Jup*^–/–^ mice. As observed before (46), DSG2 was less expressed in the intercalated discs (ICDs) of *Jup*^–/–^ mice compared with *Jup*^+/+^ mice (Figure 6, A and D). No changes in staining intensity or ICD lengths between mediator-treated and untreated slice cultures were found in murine cardiac slice cultures from *Jup*^+/+^ or *Jup*^–/–^ mice. Nonetheless, both DP and DSG2 staining were broader after EGFR or SRC inhibition at ICDs of *Jup^+/+^* mice (Figure 6, A–C). Interestingly, in cardiac slices obtained from *Jup*^–/–^ mice, only DSG2 staining width was increased after EGFR or SRC inhibition, whereas changes in DP staining width were not observed (Figure 6, D–F). Taken together, immunostaining showed increased DSG2 and DP localization at cellular junctions after EGFR or SRC inhibition.

### EGFR or SRC inhibition promotes desmosomal assembly in HL-1 cardiomyocytes.

**Figure 7:**
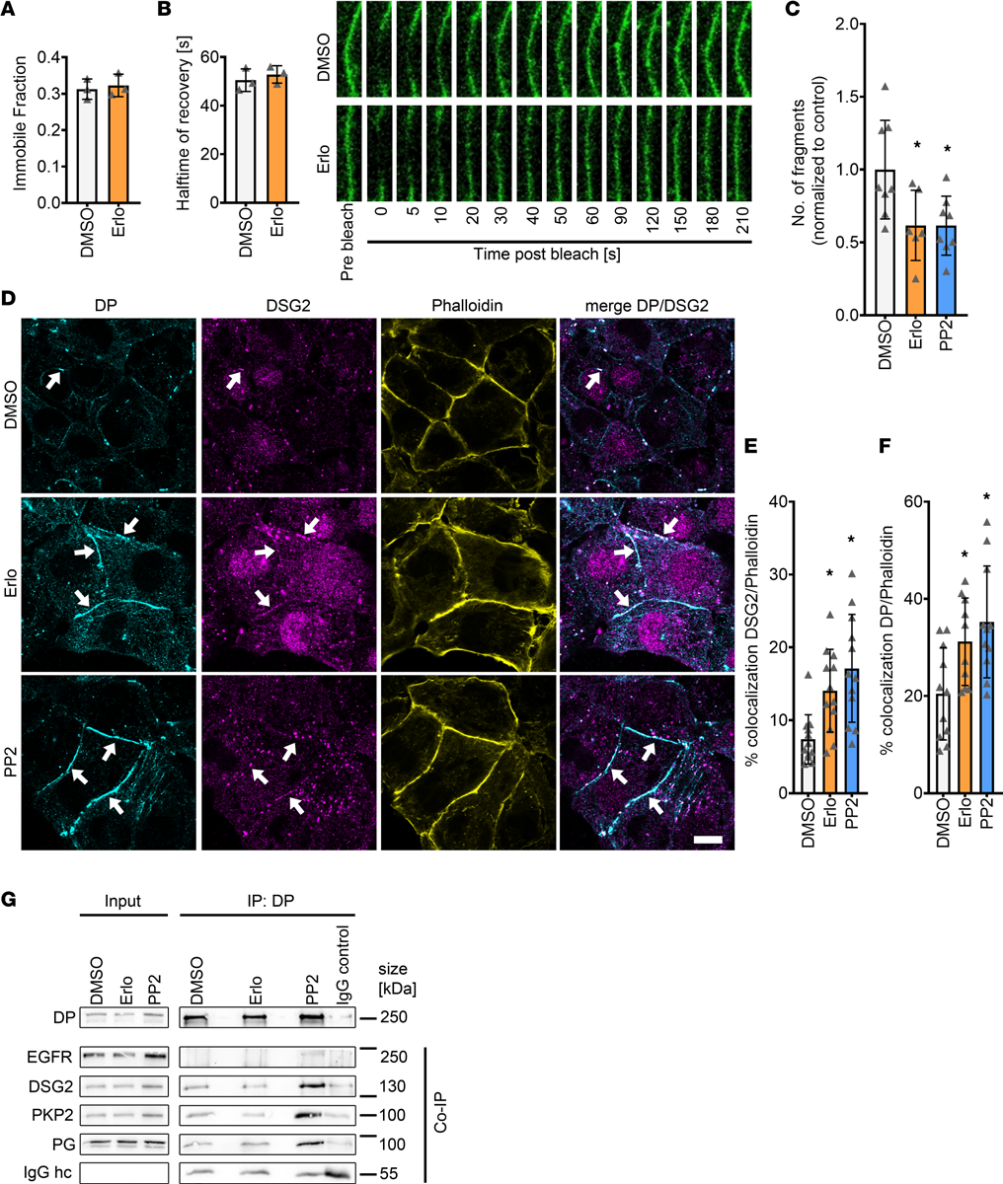
Increased recruitment of DP and DSG2 toward the cell membrane is achieved by enhanced desmosome assembly. (**A** and **B**) Fluorescence recovery after photobleach (FRAP) measurements in HL-1 cardiomyocytes transfected with DSG2-GFP and treated with DMSO or erlotinib. Measurements were performed 60-120 minutes posttreatment immobile fraction (**A**) and halftime of recovery (τ) (**B**). *P ≤ 0.05, unpaired Student’s *t* test, *n* = 4 independent experiments. (**C**) Dispase-based dissociation assay in HL-1 cardiomyocytes after 90 minutes of Ca^2+^ depletion and subsequent Ca^2+^ repletion together with erlotinib or PP2. *P ≤ 0.05, 1-way ANOVA with Holm-Sidak correction, *n* = 6 independent experiments. (**D**) Immunostaining of DP and DSG2 in HL-1 cardiomyocytes with phalloidin as membrane marker after 90 minutes of Ca^2+^ depletion and subsequent Ca^2+^ repletion together with erlotinib or PP2. White arrows indicate areas of increased DP or DSG2 recruitment to the membrane. Scale bar: 10 μm. (**E** and **F**) Quantification of colocalization of DP (**E**) or DSG2 and phalloidin (**F**). *P ≤ 0.05, 1-way ANOVA with Holm-Sidak correction, *n* = 4 independent experiments. Each data point represents 1 area. (**G**) Immunoprecipitation of DP from HL-1 lysates, and coimmunoprecipitation of EGFR, DSG2, PKP2 and PG; IgG hc served as loading control. *n* = 4–5 independent experiments.

Since the localization of DSG2 at cell borders was increased after EGFR or SRC inhibition, we investigated whether EGFR inhibition alters DSG2 mobility. To address this, we transfected HL-1 cardiomyocytes with a DSG2-GFP construct and performed fluorescence recovery after photobleach (FRAP) experiments. We neither found changes in the halftime of recovery (τ), nor in the immobile fraction after EGFR inhibition (Figure 7, A and B). Furthermore, we did not observe significant protein localization changes between cytosolic and membrane-bound fractions in Triton X-100 assays (Supplemental Figure 5, A and B). Therefore, we next studied desmosomal assembly and performed a Ca^2+^-switch assay by depleting cells of Ca^2+^ for 90 minutes, using the Ca^2+^ chelator EGTA to disrupt desmosomes that reassemble upon Ca^2+^ repletion. We performed dispase-based dissociation assays after changing the medium to Ca^2+^-containing medium (Ca^2+^-switch assay) together with erlotinib or PP2. EGFR or SRC inhibition led to increased cellular cohesion after a Ca^2+^ switch, indicating the possibility of increased desmosomal assembly (Figure 7C and Supplemental Figure 5C). Indeed, immunostaining for DP and DSG2 revealed that both EGFR and SRC inhibition led to enhanced recruitment of DP and DSG2 to the cell borders after the Ca^2+^ switch in HL-1 cardiomyocytes (Figure 7, D–F). These data confirm that EGFR and SRC inhibition led to enhanced desmosomal assembly. To further understand whether the observed demosome assembly occurs via changes in desmosomal protein interactions, we performed immunoprecipitation of DP after EGFR and SRC inhibition, which revealed no significant changes in the desmosomal complex (Figure 7G and Supplemental Figure 5D).

### Positive adhesiotropy induced by EGFR inhibition is mediated by ROCK.

**Figure 8:**
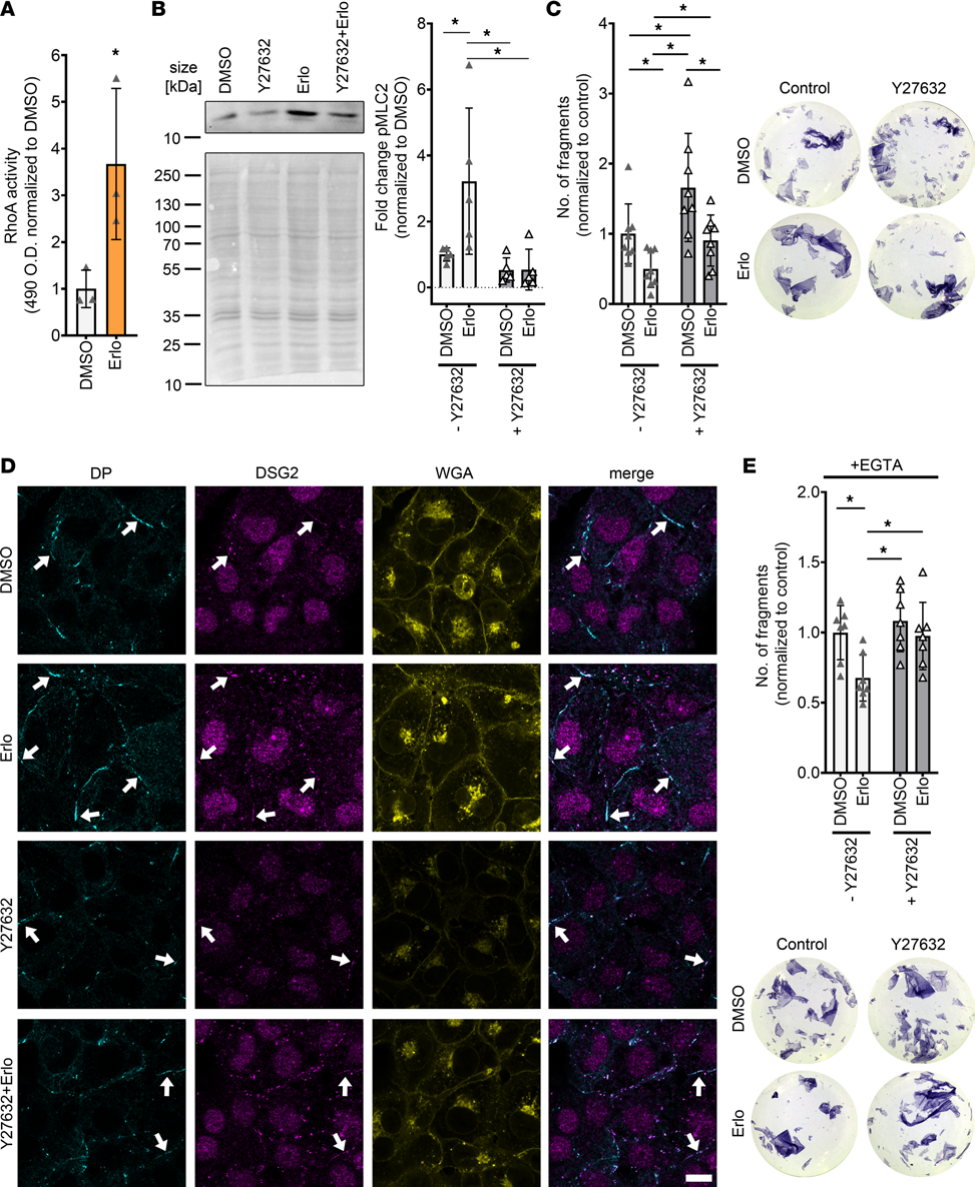
Erlotinib-enhanced cardiomyocyte cohesion and desmosomal assembly are mediated by ROCK in HL-1 cardiomyocytes. (**A**) RhoA G-LISA showing enhanced RhoA activity upon EGFR inhibition by erlotinib. *P ≤ 0.05, unpaired Student’s *t* test, *n* = 3. (**B**) Representative Western blot showing phosphorylation of the ROCK target MLC2 upon erlotinib treatment. *P ≤ 0.05, 2-way ANOVA with Holm Sidak’s multiple comparison test, *n* = 6 independent experiments. (**C**) Dispase-based dissociation assay in HL-1 cardiomyocytes, after treatment with erlotinib with and without Y27632. Y27632 was added 30 minutes prior to erlotinib incubation for 60 minutes, with representative pictures of the wells. *P ≤ 0.05, 2-way ANOVA with Holm Sidak’s multiple comparison test, *n* = 8 independent experiments. (**D**) Immunostaining of DP and DSG2 in HL-1 cardiomyocytes with WGA as membrane marker after erlotinib treatment with and without Y26732, as in **C**. White arrows indicate areas of increased DP or DSG2 recruitment to the cell membrane. Scale bar: 10 μm. (**E**) Dispase-based dissociation assay in HL-1 cardiomyocytes after 90 minutes of Ca^2+^ depletion and treatment with erlotinib with and without Y26732 with representative pictures of the wells. *P ≤ 0.05, 2-way ANOVA with Holm-Sidak’s multiple comparison test, *n* = 7 independent experiments.

We next aimed to unravel potential mechanisms of EGFR inhibition–mediated positive adhesiotropy. Since the EGFR signaling cascade involves several different kinases, we performed a multiplex kinase activity array (PamGene Kinase assay) utilizing HL-1 cardiomyocytes. Here we compared kinase activity in erlotinib-treated samples with control samples after 15, 30, and 60 minutes of incubation (Supplemental Table 1). After 30 minutes of EGFR inhibition, we observed an increase in PCTAIRE2, also known as cyclin dependent kinase 17 (CDK17), Rho-associated protein kinase 1 (ROCK1), and protein kinase N1 (PKN1) activity. Since Rho family members are known to be involved in cellular cohesion (47–49), and PKN1 is an effector kinase of RhoA (50, 51), which is upstream of ROCK, we focused on ROCK. To assess whether ROCK is activated upon EGFR inhibition, we performed RhoA G-LISA, which revealed enhanced RhoA activity upon EGFR inhibition (Figure 8A). Furthermore, we analyzed MLC2 phosphorylation, which is a downstream target of ROCK and observed increased MLC2 phosphorylation upon EGFR inhibition (Figure 8B). Indeed, inhibition of ROCK by Y27632 decreased cellular cohesion in HL-1 cardiomyocytes and prevented erlotinib-induced positive adhesiotropy in HL-1 cardiomyocytes (Figure 8C), indicating that ROCK is involved in cardiomyocyte cohesion. We then performed immunostainings and found that, upon ROCK inhibition, erlotinib did not enhance DP and DSG2 staining at the cell borders (Figure 8D and Supplemental Figure 6, A and B). To further characterize whether ROCK is involved in desmosome assembly, we performed dispase-based dissociation assays after a Ca^2+^ switch in HL-1 cardiomyocytes. ROCK inhibition alone did not decrease cellular cohesion after a Ca^2+^ switch, whereas EGFR inhibition–mediated positive adhesiotropy was still prevented (Figure 8E).

Taken together, these data suggest that ROCK is involved in the EGFR inhibition–induced positive adhesiotropy and EGFR inhibition–mediated enhanced desmosomal assembly.

## Discussion

**Figure 9:**
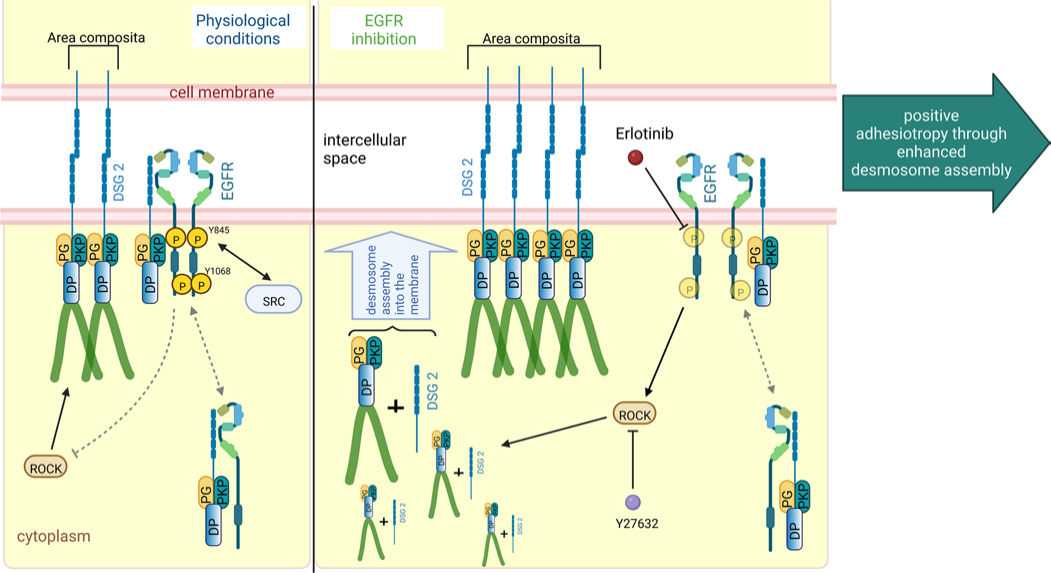
Schematic overview of EGFR inhibition–mediated positive adhesiotropy. EGFR exists in complex with DSG2, along with DP, PG, and PKP2, either at the cell borders or in the cytoplasm. Under physiological conditions, ROCK is necessary for basal cardiomyocyte cohesion. EGFR inhibition by erlotinib leads to ROCK activation, which results in positive adhesiotropy via enhanced desmosome assembly and area composita length. Inhibition of ROCK completely abrogates erlotinib-mediated positive adhesiotropy.

AC is a disease with complex pathogenesis, which at least in part is caused by disturbed desmosome turnover (52). The current therapeutic options for patients with AC include changes in lifestyle, treatment with antiarrhythmic drugs, catheter ablation, implantable cardiac defibrillators, and ultimately heart transplantation (28). However, in patients carrying mutations in genes coding for proteins of the desmosomal complex, lifestyle changes, such as restraining from physical endurance activities and β-blocker therapy, are most commonly used (28). In our previous work, we discovered adrenergic signaling via PKA-mediated PG phosphorylation at serine 665 as a driving force for DSG2-mediated enhanced cardiomyocyte cohesion, which we termed positive adhesiotropy (30, 31, 33). This indicated that cardiomyocyte adhesion at ICDs is precisely regulated and can be enhanced via drugs. Because EGFR inhibitors are used in clinics for the treatment of several cancers (8, 9), in this work, we characterized the role of EGFR on cardiomyocyte cohesion and the efficacy of erlotinib to modulate adhesion. Indeed, we show that inhibition of EGFR and its effector molecule, SRC, in HL-1 cardiomyocytes and murine cardiac slice cultures from *Jup*^+/+^ or *Jup*^–/–^ mice enhanced cardiomyocyte cohesion, paralleled by increased desmosome assembly into the area composita (Figure 9).

As a first step, we analyzed the expression of EGFR in *Jup*^+/+^ or *Jup*^–/–^ mice hearts and found an increase in EGFR expression in the hearts of *Jup*^–/–^ mice. Furthermore, we observed increased EGFR levels in the human heart atrial appendage obtained during heart transplantation from patients with AC or DCM. However, it must be noted that the ventricular myocardium is more affected in AC than atria. Nevertheless, we also found upregulation of EGFR in another murine AC model, *Pkp2^–/–^* mice, reinstating that EGFR overexpression in murine AC was not a one-off effect. To our knowledge, this is the first instant of finding EGFR overexpression in AC. In fact, EGFR activation and signaling was shown to promote TGF-β–dependent renal fibrosis (53, 54). Since in AC, replacement of myocardium with fibrotic tissue is a common phenomenon (28), we believe that the observed EGFR overexpression might be related to fibrosis observed in *Jup*^–/–^ mice (31, 33, 46). It has been shown that, upon treatment with the EGFR inhibitor gefitinib, inflammation and hypertrophy caused by chronic treatment with the adrenergic mediator isoprenaline was ameliorated in mice (55, 56). Moreover, increased EGFR expression was positively correlated with the size of atherosclerotic plaques (57). However, mice with a strong downregulation of EGFR in the heart have severe hypertrophy and hypotension (15).

We used erlotinib or PP2 to inhibit EGFR or SRC kinases, respectively, and focused more on erlotinib, since it is a clinically approved drug. Dispase-based dissociation assays using erlotinib or PP2 in HL-1 cardiomyocytes and murine cardiac slice cultures from *Jup*^+/+^ or *Jup*^–/–^ mice revealed that both inhibitors enhance cardiomyocyte cohesion. Similar to our findings in this study, EGFR inhibition has been shown to increase cellular cohesion in squamous cell carcinomas (40), whereas in keratinocytes, EGFR inhibition protected against DSG3 autoantibody–induced loss of keratinocyte adhesion (39). Immunoprecipitation revealed EGFR along with PKP2, DP, and PG as being in a complex with DSG2. In intestinal epithelial cells, as well as in a cell-free AFM setting, an interaction of DSG2 and EGFR has been observed (41). However, to our knowledge, this complex was not known before in cardiomyocytes. It has been well demonstrated that desmosomes in the epithelia or ICDs of the cardiomyocytes not only provide cellular cohesion but can also be part of a signaling hub (35, 38, 58, 59), thus disrupting EGFR signaling in this context might lead to changes at the desmosome.

On a single-molecule level in an AFM setting, we found that binding events mediated by DSG2, but not DSC2 and N-CAD, were increased after erlotinib treatment in HL-1 cardiomyocytes. Increased binding frequency at the cell borders indicates an increased binding partner localization toward the cell borders. However, active EGFR may disturb the interaction possibility of the binding partner with the DSG2-coated AFM-tip. The interaction might be restored upon EGFR inhibition. In a cell-free AFM setting, DSG2 not only interacts homophilically with DSG2 but also interacts heterophilically with DSC2 (60). In the AFM setting, only interactions with “free” proteins can be measured. Since we observed an increase of DSG2 at the cell borders upon EGFR inhibition, we suppose that the increased binding frequency at cell borders, in line with our results from the Ca^2+^-switch assays, indicate increased desmosomal assembly, where freshly assembled desmosomal precursors are available for interaction with the opposite cell DSG2 or DSC2. At the same time, the decrease of DSC2 binding frequency might indicate that either DSC2 or its binding partners are not available for interactions with the DSC2-coated tips, especially at the cell surface. Since we did not observe any significant changes in the DSC2 binding frequency at the cell border, we assumed that the observed decrease in DSC2 binding frequency at the cell surface was not of great concern.

When we knocked down *Egfr* by siRNA, SRC inhibition was still effective in inducing positive adhesiotropy, indicating that the SRC inhibition–mediated effect on cardiomyocyte cohesion is either downstream or at least in part independent of EGFR. However, Western blot analyses revealed that PP2 also caused a significant decrease in EGFR phosphorylation at Y845 and Y1068, indicating that SRC acts upstream of EGFR. In a previous study, SRC was found close to focal adhesions partially overlapping with α-actinin and vinculin (61), indicating that SRC might affect actin-anchorage in cardiomyocytes and, thereby, could also influence desmosome assembly. Further studies are required to unravel the role of SRC and actin-anchorage in desmosome assembly in cardiomyocytes. On the other hand, we have previously shown in keratinocytes that SRC via its target cytoskeletal protein cortactin contributes to the loss of cell cohesion mediated by DSG3 autoantibodies (62). Similarly, in cardiomyocytes, SRC might also have a direct interaction with desmosomal proteins, independent of the EGFR molecule. In fact, in intestinal epithelial cells, DSG2, EGFR, and SRC were found to be in complex (41). Upon knockdown of *DSG2*, DSC2 has been shown to be upregulated in colonic adenocarcinoma cell lines (45), and this might explain why we can still observe a positive adhesiotropic effect of EGFR or SRC inhibition upon *Dsg2* knockdown. Furthermore, in previous reports, inhibition of EGFR enhanced DP expression in breast cancer cells and led to enhanced DP at the cell border in oral squamous cell carcinoma cells (40, 63). On the other hand, the remaining DSG2 upon *Dsg2* knockdown could be sufficient to induce positive adhesiotropy after EGFR inhibition. Indeed, we never observed a decrease in cell cohesion upon *Dsg2* knockdown (33, 42), further confirming that the partial presence of DSG2 can provide mechanical stability to the cells.

Furthermore, we observed an increased recruitment of DSG2 and DP to the membranes of HL-1 cardiomyocytes, increasing area compositae length. The longer area composita in HL-1 cardiomyocytes could be the cellular correlate of increased thickness of DSG2 and DP staining in murine cardiac slices. A similar reorganization of ICDs has already been observed in electron microscopy upon adrenergic signaling, which also induces positive adhesiotropy in cardiomyocytes (46). We found that the recruitment of DSG2 and DP into the cellular junctions was not caused by increased mobility; rather, it was caused by an enhanced desmosomal assembly, which is supported by increased DES insertions into DP. Interestingly, in *Jup*^–/–^ mice, we observed changes in DSG2 but not DP localization at the ICD, indicating that the effect of EGFR and SRC inhibition in intact cardiac tissue is at least in part mediated by PG. Indeed, it was shown previously that, in *Jup*^–/–^ keratinocytes, steady-state protein levels and recruitment of desmosome components to the membrane were reduced (64).

Mechanistically, PG-phosphorylation at tyrosine residues was shown to be involved in EGFR-induced loss of cell adhesion, which decreased association of PG to DP (65) and triggered translocation of PG and DP from the membrane toward the cytoplasm (44, 65). Furthermore, EGF-induced phosphorylation of PG could be prevented by EGFR inhibition (40). However, we did not consistently observe increased coimmunoprecipitation of PG and PKP2 with DP in HL-1 cardiomyocytes after inhibition of EGFR or SRC.

Similar to our findings in this study, in keratinocytes, inhibition of ROCK increased cellular migration and decreased cellular cohesion (66, 67). In corneal epithelial cells, inhibition of ROCK led to a translocation of E-Cadherin to the cytoplasm (68). Apart from that, using an in silico model, it has been suggested that ROCK plays a role in the pathogenesis of AC (69). Furthermore, inhibition of ROCK during early cardiac development did lead to AC in a murine model with a reduced localization of PG at the ICD (70). An involvement of RhoA, which is upstream of ROCK, in functional desmosomal assembly has been described in squamous carcinoma cell lines (71). In cardiomyocytes derived from induced pluripotent stem cells (iPSCs) of a patient with AC carrying a *PKP2* mutation, upregulation of genes inhibiting the ROCK signaling pathway were found. Furthermore, inhibition of ROCK over the course of 14 days led to a decrease in cellular cohesion and decreased PKP2 localization at the membrane in WT iPSC-derived cardiomyocytes, whereas after 28 days of ROCK inhibition, adipocyte-like cells were observed (72). Here, we observed that even short-term ROCK inhibition for 90 minutes decreased cellular cohesion but did not alter DSG2 or DP localization to the cell borders significantly in HL-1 cardiomyocytes, though a nonsignificant decreasing trend could be observed. Nonetheless, ROCK inhibition decreased erlotinib-induced DP and DSG2 localization to the cell borders. Furthermore, after a Ca^2+^ switch, ROCK inhibition alone did not alter the cellular cohesion, whereas it prevented the enhanced desmosomal assembly induced by erlotinib. We, therefore, presume that erlotinib-mediated positive adhesiotropy in cardiomyocytes requires ROCK. Erlotinib-mediated positive adhesiotropy might also be caused by mechanisms other than enhanced desmosomal assembly. However, since we observed an increase in protein localization of DSG2 and DP at the membrane after Ca^2+^ switch, we believe that erlotinib enhances desmosomal assembly. We suppose that a possible effect of ROCK inhibition on cellular cohesion after a Ca^2+^ switch was below the detection limit of dissociation assays. These results warrant further studies to understand the role of EGFR and ROCK in the regulation of cardiomyocyte cohesion and eventually in the pathomechanisms of AC. Furthermore, the interaction of ROCK and desmosomal components should be assessed.

Based on our results, we anticipate that EGFR inhibition can be beneficial in the setting of AC. Furthermore, unlike other EGFR inhibitors, reports of adverse cardiac effects after erlotinib treatment are very rare (16, 73–75). Taken together, we conclude that inhibiting EGFR, thereby enhancing desmosome assembly via ROCK, stabilizes desmosome integrity and cardiomyocyte cohesion. However, further studies, investigating the in vivo effect of EGFR inhibition utilizing AC mouse models as well as the effects on electromechanical coupling are needed, which are beyond the scope of our current study. Nevertheless, we believe that our study is a first step that paves the way for future studies targeting EGFR inhibition or ROCK activation by erlotinib as a treatment option for AC.

## Methods

### Cell lines.

HL-1 cells, which are immortalized female murine atrial myocyte cells, provided by William Claycomb (LSU Health Sciences Center, New Orleans, Louisiana, USA), were cultured in supplemented medium as described before (33). Cells were cultured in medium containing norepinephrine, while for experiments, medium without norepinephrine was used.

### Murine cardiac slice culture from Jup^+/+^ and Jup^–/–^ mice.

Previously characterized 12-week-old C57BL6/J WT and cardiomyocyte specific *Jup*^–/–^ mice were used for experiments (31, 42). In brief, mice with loxP sites flanking exon 1 of the junctional PG gene (*Jup*) on both alleles (*Jup^tm1.1Glr/J^* mice, The Jackson Laboratory) were bred with mice heterozygous for loxP sites in *Jup* with an expression of the recombinase Cre under the control of cardiomyocyte-specific α myosin heavy chain promoter (Myh6), which were generated by crossing with B6.FVB-Tg(Myh6-cre)2182Mds/J mice (The Jackson Laboratory).

Murine cardiac slice cultures were obtained as described before (33) with minor adjustments. In brief, after anesthetizing mice with isoflurane, mice were sacrificed by cervical dislocation, and the hearts were collected, put into precooled slicing buffer, embedded in low-melt agarose, and sliced with a vibratome (LeicaVT1200S vibratome) into 200 μm sections. For treatments, consecutive slices were used to minimize slice-to-slice variability. After respective treatments, slices were processed for lysate preparation, dispase-based dissociation assays, and immunostainings. For immunostainings, slices were embedded in NEG-50 frozen section medium and stored at –80°C until further processing.

### Lysates from Pkp2^–/–^ mice.

Protein lysates from hearts of homozygous cardiac-restricted *Pkp2-*KO mice (*Pkp2*^–/–^) and respective age-matched Cre-negative littermates were provided by Brenda Gerull from an unpublished mouse model. *Pkp2* KO was generated by an intercross of C57BL/B6J.129.*Pkp2*^tm1Mdcb^ (*Pkp2*^fl/fl^) mice (76) (provided by the Max Delbrück Center for Molecular Medicine, Berlin) with α-MyHC–Cre mice (77). Mice heterozygous for the *Pkp2* deletion were then mated with *Pkp2*^fl/fl^ animals to obtain homozygous KOs.

### Human heart lysates for Western blot.

Human heart samples from patients diagnosed with DCM or AC were obtained during heart transplantation, snap-frozen in liquid nitrogen, and processed for Western blot analysis by lysing in SDS-lysis buffer.

### Mediators.

Samples were treated with mediators for 90 minutes. Erlotinib (Santa Cruz Biotechnology Inc., sc-202154) was used as an EGFR inhibitor at a final concentration of 2.5 μM. PP2 (Merck, 529573) was used to inhibit the EGFR signaling pathway by inhibiting SRC at a final concentration of 10 μM. Y27632 was used to inhibit ROCK at a final concentration of 10 μM (Tocris, 1254). When a Ca^2+^ switch was performed, cells were treated with 5 mM EGTA (VWR, 0732) for 90 minutes. Then, medium was changed back to Ca^2+^-containing medium for 90 minutes, and mediators were added at the same time.

### Lysate preparation.

For Western blot analyses, lysates were prepared in SDS lysis buffer with protease and phosphatase inhibitors (cOmplete Protease Inhibitor Cocktail, Roche; CO-RO and PhosStop, Roche, PHOSS-RO). In the case of HL-1 cardiomyocytes, the medium was removed after treatments and kept on ice, washed with PBS, scraped into SDS lysis buffer, and sonicated. When working with murine cardiac slice cultures, slices were treated, washed with TBS, and snap-frozen in liquid nitrogen. To prepare the tissue lysates, SDS lysis buffer was added and the samples were transferred into gentleMACS M-tubes (Miltenyi Biotec, 130-093-236) and dissociated using the protein_01_01 program of the gentleMACS OctoDissociator (Miltenyi Biotec, 130-095-937).

For coimmunoprecipitation experiments, cells were cultured in T75 flasks. After treatments, RIPA lysis buffer (10 mM Na_2_HPO_4_, 150 mM NaCl, 1% Triton X-100, 0.25% SDS, 1% sodium deoxycholate [pH 7.2]) containing protease and phosphatase inhibitors was added to the cells and incubated for 30 minutes on ice on a rocking platform. The lysate was then transferred to gentleMACS M-tubes and dissociated using the protein_01_01 program of the gentleMACS OctoDissociator.

For Triton assays, cells were washed with ice-cold PBS, and cells were kept on ice on a rocking platform in Triton buffer (0.5% Triton X-100, 50 mM MES, 25 mM EGTA, 5 mM MgCl_2_ [pH 6.8]) with protease and phosphatase inhibitors for 20 minutes. After that, the lysate was transferred to Eppendorf tubes and centrifuged at 4°C for 10 minutes at 15,000*g* in an Eppendorf 5430R centrifuge. The supernatant containing the Triton-soluble proteins was transferred to a new reaction tube, while the pellets were washed with Triton buffer, dissolved in SDS lysis buffer, and sonicated.

For the PamGene Kinase assay, after the respective treatments cells were washed twice with ice-cold PBS and lysed into M-PER lysis buffer (Thermo Fisher Scientific, 78503) supplemented with Halt Protease and Phosphatase inhibitors (Thermo Fisher Scientific, 87786 and 78420). The lysate was transferred to Eppendorf tubes and centrifuged at 4°C for 15 minutes at 15,000*g* in an Eppendorf 5430R centrifuge, and the supernatant was aliquoted into fresh tubes, snap-frozen in liquid nitrogen, and stored at –80°C until further analysis.

For all assays, protein concentration was measured using the Pierce Protein Assay Kit (Thermo Fisher Scientific, 23225) according to the manufacturer’s protocol.

### Western blot analyses.

Lysates were denatured in Laemmli buffer and boiled for 10 minutes at 95°C, before being loaded on SDS-PAGE gels. The PageRuler Plus Prestained Protein Ladder (Thermo Fisher Scientific, 26620) was used as a marker. After electrophoresis, proteins were transferred to a nitrocellulose membrane (Thermo Fisher Scientific, LC2006) using the wet-blot method. Membranes were then blocked in 5% milk/TBST, in case of anti–phospho-EGFR antibodies (Cell Signaling Technology, 2231) in 5% BSA/TBST, or in case of anti-DP antibodies in ROTI Block (Carl Roth, A151.2). For Western blots with lysates from murine tissue samples, as well as Triton blots, the NoStain Protein Labeling Reagent (Thermo Fisher Scientific, A44449) was used as loading control according to the manufacturer’s protocol. For human cardiac lysates, 0.2% Ponceau S in 3% acetic acid was used as loading control. Ponceau S was also used as loading control for phospho-MLC2 blots due to a lack of functional total MLC2 antibody. The following primary antibodies were added overnight at 4°C on a rocking platform: anti-DES (Abcam, ab32362), anti-DP (Progen, 61003), anti-DSG1/2 (Progen, 61002), anti–phospho-EGFR Y845, anti–phospho-EGFR Y1068 (Cell Signaling Technology, 2234), anti-EGFR (Santa Cruz Biotechnology Inc., sc-373746 [for HL-1 cardiomyocytes], and Abcam, ab52894 [for murine tissue]), anti–phospho-ERK1/2 (E-4; Santa Cruz Biotechnology Inc., sc-7383), anti-ERK (Cell Signaling Technology, 9102), anti–phospho-MLC2 (Cell Signaling Technology, 3671), anti–N-CAD (BD Biosciences, 610921), anti-PG (PG5.1; Progen, 61005), anti-PKP2 (Progen, 651167), and anti–α-tubulin (DM1A; Abcam, ab7291). After washing, membranes were incubated with species-matched, HRP-coupled secondary antibodies (Dianova, 111-035-045 or 115-035-068) diluted in TBST or 5% milk/TBST in case of phospho-EGFR antibodies, and developed using an iBright FL1500 (Thermo Fisher Scientific) developer with SuperSignal West Pico PLUS Chemiluminescent Substrate (Thermo Fisher Scientific, 34577). All primary antibodies were used at 1:1,000 dilutions, apart from phospho-EGFR and EGFR antibodies (1:500), phospho-PG (1:20), anti-PKP2 (1:25), and anti–α-tubulin (1:4,000). Quantification was performed using the Image Studio Lite v.5.2 or the iBright Analysis Software (Invitrogen). See supplemental material for complete, unedited blots.

### Dissociation assays.

Dissociation assays in HL-1 cardiomyocytes were performed as described before (33). In brief: after treatment, medium was removed and Liberase-DH (Sigma-Aldrich, LIBDH-RO) was added, followed by dispase II (Sigma-Aldrich, D4693). Cells were incubated at 37°C, 5% CO_2_, until the cell monolayers detached from the well bottoms. Then, the enzyme mix was carefully replaced by HBSS, and mechanical stress was applied by shaking at 1.31*g* for 5 minutes on an orbital shaker (Stuart SSM5 orbital shaker). For better visibility, MTT (Sigma-Aldrich, M5655) was added to the cells and images were taken to count the number of fragments. The amount of fragmentation serves as an indirect measurement of intercellular cohesion, as fewer fragments suggest a stronger cellular cohesion.

Dissociation assays in murine cardiac slices were performed similarly to dissociation assays in HL-1 cardiomyocytes. However, in this case, Liberase-DH and dispase II were added simultaneously for 30 minutes. MTT was added, and equal mechanical stress was applied to all samples of an experiment using an electrical pipette. Then, the content of each well was filtered using a 70 μm nylon membrane (PluriSelect, 43-10070-60) and transferred to a gridded 96-well plate. Pictures of the well were taken and stitched together using the AutoStich software (78). For quantification, only the number of viable dissociated cardiomyocytes was counted, distinguishable by their rod shape and the MTT staining. To control variations due to different slice size or location in the ventricle, consecutive slices for control and treatments were used, and treatments were normalized to the respective control slice to reduce slice-to-slice and mouse-to-mouse variability.

### Immunostainings in HL-1 cardiomyocytes and murine cardiac slices.

Cells were seeded on coverslips and fixed with paraformaldehyde (PFA) after respective treatments. Sections of murine cardiac slices (7 μm thick) were cut with a cryostat (HM500OMV, Microm); sections were transferred to glass slides and adhered by warming up to 38°C. After PFA fixation, the samples were permeabilized with 0.1% Triton X-100 and blocked with BSA and normal goat serum (BSA/NGS). Primary antibodies diluted in PBS were added and incubated overnight at 4°C in a wet chamber. The following primary antibodies were used: anti-DES (Abcam, ab32362), anti-DP (Progen, 61003), anti-DSG2 (Progen, 610121), anti-DSG2 (Thermo Fisher Scientific, PA5-79171), and anti–N-CAD (BD Biosciences, 610921). When working with HL-1 cardiomyocytes, PBS was used for washing steps, while, for murine tissue samples, 50 mM NH_4_Cl in PBS was used. After washing, species-matched, fluorophore-coupled secondary antibodies (anti–mouse Alexa Fluor 488 [Dianova, 115-545-003], anti–mouse Cy3 [Dianova, 115-165-164], or anti–rabbit Cy5 [Dianova, 111-175-144]) were added for 60 minutes. In the case of wheat germ agglutinin (WGA; Thermo Fisher Scientific, W11261) or phalloidin staining (Thermo Fisher Scientific, A12379), the dye was added together with the secondary antibodies. WGA was used at 2 μg/mL and phalloidin at 130 nM concentrations. During the last 10 minutes of secondary antibody incubation, DAPI (Sigma Aldrich, D9542, 0.5 μg/mL) was added. After washing, coverslips were mounted on microscope slides using NPG (Sigma-Aldrich, P3130). Slides were analyzed using a Leica SP5 II confocal microscope (Leica) with a 63***×*** oil objective with a numerical aperture of 1.4 using Immersol 518F (Zeiss). Images were acquired at room temperature with the LAS-AF software. Z-scans were performed at 0.25 μm thickness spanning the whole cell volume. Colocalization analyses were performed as described before (33). In brief, the membrane region was marked as a region of interest (Supplemental Figure 7A); in this region, the amount of stained pixels in the other channel, where colocalization was to be quantified, was measured, and a ratio of colocalization at the membrane was calculated. Measurement of staining width in immunostainings was performed in murine cardiac slice cultures, exemplified in Supplemental Figure 7B.

For STED immunostainings, a slightly modified staining protocol was used: instead of washing with PBS, cells were washed with 50 mM NH_4_Cl in PBS. Primary antibodies were diluted in BSA/NGS, and the following secondary antibodies were used: STAR RED anti-rabbit (Abberior, STRED-1002) and Alexa Fluor 594 anti-mouse (Abcam, ab150120), both 1:200 dilution. Slides were mounted with ProLong Diamond Antifade (Thermo Fisher Scientific, P36961). Samples were analyzed at room temperature using a STED expert line microscope from Abberior with a 100***×*** oil objective with a numerical aperture of 1.4 using IMMOIL-F30CC (Olympus). Images were acquired with a DMK33G274 camera and the Imspector software. For quantification, areas with a “railroad-like” staining of DP were considered as area composita. To quantify DES insertions into the area composita, the number of spots where DES filaments were inserted into the area composita were counted.

### Immunoprecipitation.

For pulldowns, 1 μg of respective antibodies was added to 1.5 mg of protein lysate overnight and kept at 4°C on a spinning wheel. To rule out unspecific antibody or bead binding, IgG control samples were prepared in parallel. Therefore, the same amount of protein lysate was used as for pulldowns; however, instead of primary antibody, 1 μg of normal mouse IgG (Merck, 12-371) was added to the samples. Pulldowns were performed with Protein G Dynabeads (Thermo Fisher Scientific, 10004D) by adding the lysate-antibody mix for 1 hour at 4°C on a spinning wheel to beads prewashed in RIPA buffer with protease and phosphatase inhibitors. Washing steps were performed using a magnetic rack. Then, beads were washed twice with wash buffer 1 (50 mM Tris-HCl, 150 mM NaCl, 0.1 mM EDTA, 0.5% Tween 20 [pH 7.5]), three times with wash buffer 2 (100 mM Tris-HCl, 200 mM NaCl, 2M urea, 0.5% Tween 20 [pH 7.5]), and twice with 1% Triton X-100 in PBS. All wash buffers were supplemented with protease and phosphatase inhibitors. After the last wash step, beads were resuspended in Laemmli buffer and denatured for 10 minutes at 95°C; the lysate was separated from the beads using a magnet and loaded on a SDS-PAGE gel for Western blot analyses. A total of 20 μg of total lysate was used as input control for the Western blots.

### AFM.

AFM measurements were performed as described before (31, 42) using a Nanowizard III AFM (JPK instruments) with an optical fluorescence microscope (Axio Observer D1, Carl Zeiss) at 37°C. Cantilevers (MLCT AFM Probes, Bruker) were functionalized with recombinant proteins as described elsewhere (79). The following recombinant proteins were used: DSC2-Fc, DSG2-Fc (both self-made by Protein A purification of cell culture supernatants from overexpressing CHO cells, as described in ref. 80) or recombinant N-CAD-Fc (R&D Systems, 6626-NC-50). The canonical pyramidal-shaped D-tip was used. The thermal noise method (81) was used to determine the spring constants of the tips in order to obtain correct values for unbinding forces. For experiments, HL-1 cardiomyocytes were seeded on 24 mm glass coverslips. A bright-field image of the cells of interest was acquired with a 63***×*** objective. Afterward, an overview picture of an area of interest was taken in the force-based quantitative imaging (QI) mode with the following settings: pulling speed, 50 μm/s; set point, 0.2 nN; z-length, 2.5 μm. Based on the QI, areas for adhesion measurements were chosen (Supplemental Figure 7C). Adhesion measurements were performed on cell borders as well as cell surfaces on an area of 1.5 μm × 5.0 μm using the force mapping mode with the following settings: relative set point, 0.2 nN; z-length, 2.5 μm; extension speed, 5.0 μm/s; extend and retract delay, 0.1 s. Information of approach and retraction forces was recorded in force-distance curves (FDCs). The resulting FDCs were analyzed using the JPKSPM Data processing software. Binding events are visible by a step in the FDCs, representing the force needed to rupture the binding between the functionalized tip and the binding partner on the cell. Hence, this force is the unbinding force. Unbinding forces were analyzed using the extreme fit distribution in Origin 2017 software.

### siRNA-mediated knockdown.

For siRNA-mediated knockdown, cells were transfected with a transfection reagent, composed of OptiMEM (Thermo Fisher Scientific, 31985070), RNAiMAX (Thermo Fisher Scientific, 13778150), and the ON-Target siRNA (Dharmacon; siNT, D-001810-10; si*Egfr*, L-040411-00-0005; si*Dsg2*, L-042514-01-005; si*Dsp*, L-040653-01-005) according to manufacturer protocol. Cells were transfected for 24 hours. Seventy-two hours after transfection, medium was changed again. Experiments were performed 84 hours after transfection. Each knockdown was confirmed by Western blot.

### FRAP.

For FRAP measurements, HL-1 cardiomyocytes were seeded in ibidi 15 μ-Slide 8 Well Glass Bottom chambers (ibidi, 80827). Twenty-four hours after seeding, cells were transfected with 1 μg DSG2-GFP construct as described before (31), using TurboFect (Thermo Fisher Scientific, R0531). Images were captured with a Leica SP5 confocal microscope (Leica) with the LAS-AF software starting 60 minutes after treatment with the vehicle or erlotinib. Using the Leica FRAP Wizard, 5 pictures were taken before bleaching; then, an area of interest was bleached with 20 pulses at 100% laser intensity for 0.523 s. After that, 60 pictures were taken every second, 20 pictures were taken every 3 s, and 10 pictures were taken every 10 s. Halftime of recovery, as well as immobile fractions, were calculated using the Leica FRAP Wizard.

### PamGene kinase assay.

The serine-threonine kinase (STK) and protein tyrosine kinase (PTK) microarray assays were performed according to the manufacturer’s instructions (PTK assay on PamStation 12 User Manual, Version 3.0; Serine Threonine Kinase assay on PamStation 12 User Manual, Version 5.1) and as described earlier (82). In brief, cell lysates were combined with reaction mixtures produced with the contents of the STK reagent kit (PamGene, 32201) or PTK reagent kit (PamGene, 32112) and ampuwa ultrapure water (Fresenius Kabi) and placed on STK PamChips (PamGene, 32501) and PTK PamChips (PamGene, 32508), respectively. Cell lysates originating from the same culture passage and experimental setup (*n* = 3) were processed within the same assay run to enable accurate comparisons. FITC-conjugated anti-phosphotyrosine and anti-phosphoserine antibodies were used for visualization during and after the pumping of lysates through the 3-dimensional surface of the array. The capture of substrate phosphorylation signals was enabled by a computer-controlled CCD camera and measured repeatedly during a 1-hour kinetic protocol using the Evolve software (PamGene International B.V.). The analysis of the images was performed using the BioNavigator Software (Ver. 6.3), with the predesigned protocols “STK Image Analysis,” “PTK Image Analysis,” “STK Basic Processing,” “PTK Basic Processing,” and “Log Fold change (No Log).” Additional applications used were “Fit and apply a combat model,” “STK upstream kinase analysis,” and “PTK upstream kinase analysis.” After visual check and quality control, endpoint signal intensities minus background signals were calculated by BioNavigator for each spot, representing each kinase peptide substrate per array. Subsequently, the data were log_2_ transformed before mean replicate signal intensity within each experiment was calculated for each peptide substrate. A kinase was considered to be modulated (either activated or inhibited) if it had a mean specificity score (negative decadic logarithm of the likelihood of obtaining a higher difference between the groups when assigning peptides to kinases randomly) of 1 and a significance score (likelihood of obtaining a higher difference for random assignment of values to treatment and control groups) of 0.5. To detect changes caused by erlotinib, samples for each time point were compared with the vehicle control samples for the same time point. The mean kinase statistic (calculated by averaging the difference between the signal intensity of a sample and its control value, normalized against a pooled estimate of the SD in each sample, for each peptide assigned to a specific kinase) was used for further analysis.

### RhoA G-LISA.

RhoA G-LISA was performed using the RhoA G-LISA Activation Assay kit (Biozol, BK124), which measures cellular levels of GTP-loaded RhoA, according to the manufacturer’s instructions. Per condition, 12.5 μg protein was used. Absorbance was measured with a TECAN, Infinite 200 PRO microplate reader at 490 nm.

### Statistics.

Images were processed with Adobe Photoshop CS5, Image Studio Lite v.5.2, and ImageJ software (NIH). The illustration was created using BioRender. Statistical analyses were performed using GraphPad Prism 8. Two-tailed Student’s *t* tests or 1- or 2-way ANOVA with post hoc tests were applied after outlier removal and are explained in figure legends. Data are represented as mean ± SD. For dissociation assays as well as for quantification of Western blots in HL-1 cardiomyocytes, values were normalized to the average control value of all experiments. For dissociation assays in murine cardiac slices, the number of dissociated cells was normalized to the respective consecutive control slice of the same mouse to reduce slice-to-slice variability in slice size. Significance was assumed for *P* ≤ 0.05.

### Study approval.

The study conformed to the principles outlined in the Declaration of Helsinki and was approved by the Ethics Committee’s approval at LMU (240-12 and 21-0787 for cardiac tissue samples used for Western blots). Animal handling was in accordance with the guidelines from the Directive 2010/63/EU of the European Parliament and approved by the regional government of Upper Bavaria (Gz. ROB-55.2-2532.Vet_02-19-172, for *Jup* mice) or the local ethics committee of the government of Lower Franconia (RUF-55.2.2-2532-2-663 for *Pkp2* mice).

## Author contributions

MS, OD, PM, SM, MH, and CO acquired the data. TW and BG acquired and provided *Pkp2*^–/–^ lysates. SK, SC, and DS acquired and provided human AC and DCM samples. MS and SY analyzed the data and drafted the manuscript. All authors proofread the manuscript. RJL supervised the PamGene Kinase assay. JW and SY handled supervision of the project. JW and SY made critical revision of the manuscript for important intellectual content and designed the research.

## Supplementary Material

Supplemental data

Supplemental data set 1

## Version 1 02/16/2023

In-Press Preview

## Version 2 03/22/2023

Electronic publication
