# Supplementary material for: EGFR inhibition leads to enhanced desmosome assembly and cardiomyocyte cohesion via ROCK activation
Source: JCI Insight. 2023 Mar 22;8(6):e163763. doi: 10.1172/jci.insight.163763 (PMC10070108; doi:10.1172/jci.insight.163763)
Supplement: Supplemental data [file jciinsight-8-163763-s220.pdf]

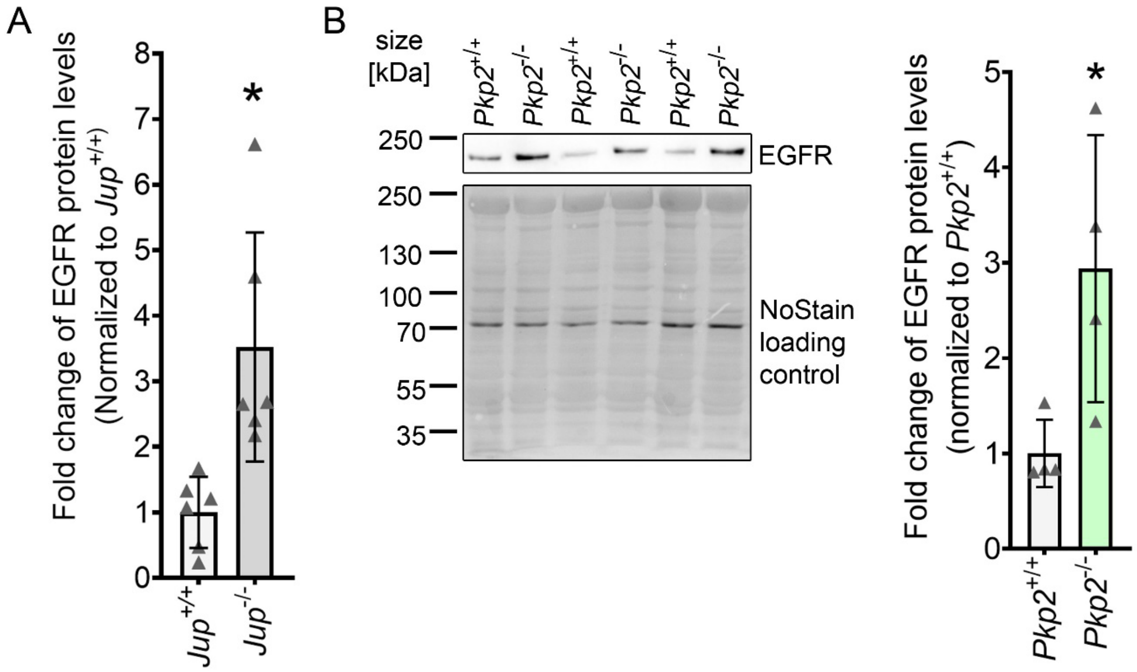

Figure S1 – Upregulation of EGFR in AC

A: Quantification of Western blots (Figure 1A) showing protein expression of EGFR in murine cardiac slices from *Jup*$^{+/+}$ and *Jup*$^{-/-}$ mice. * $p \le 0.05$ unpaired Student's *t*-test, N=6 mice**. B**: Representative Western blot showing protein expression of EGFR in heart lysates obtained from *Pkp2*$^{+/+}$ and *Pkp2*$^{-/-}$ mice. NoStain™ Protein Labeling Reagent was used as loading control. * $p \le 0.05$, unpaired Student's *t*-test, N=4 mice.

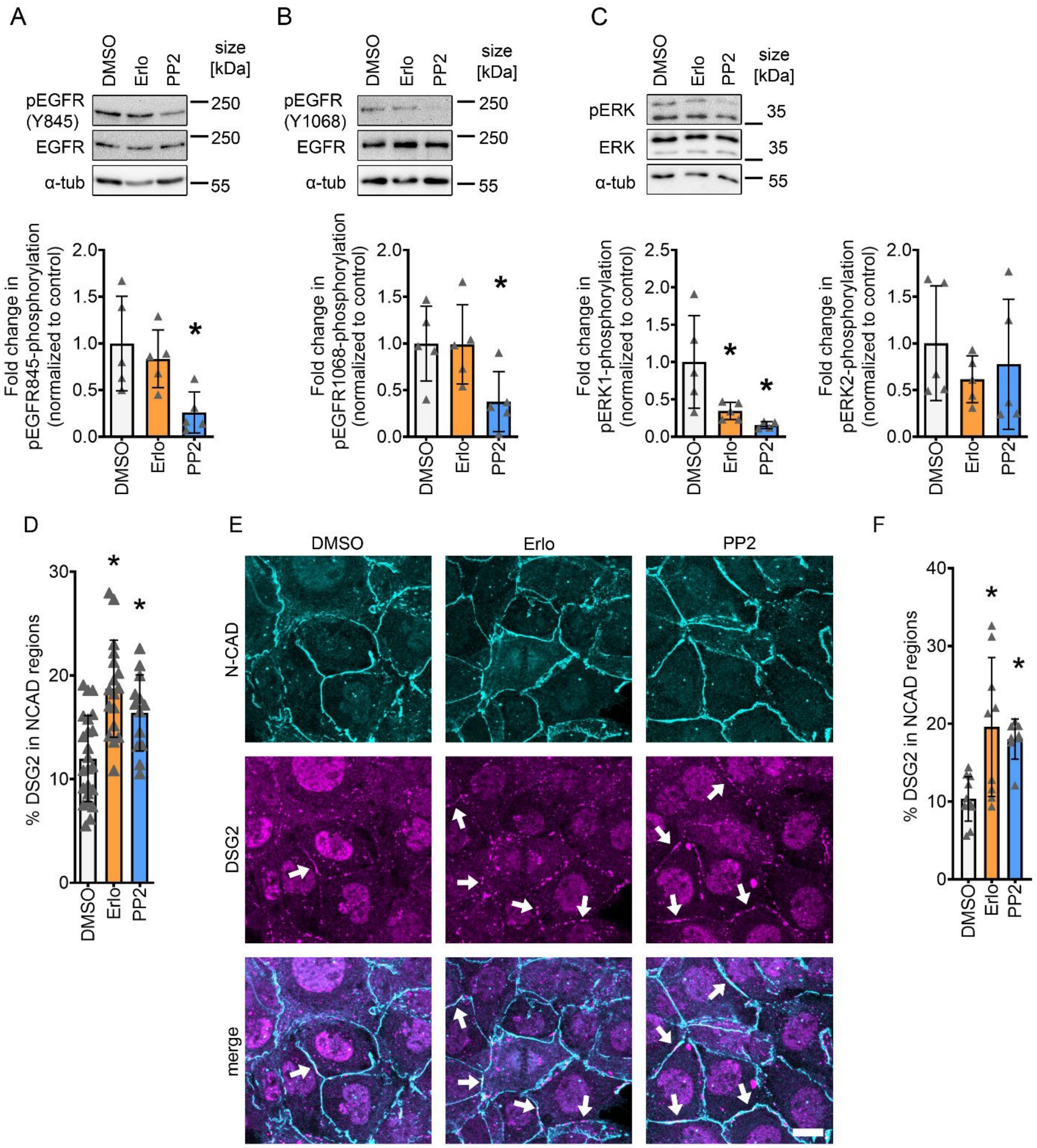

Figure S2 – confirmation of erlotinib and PP2 effect

A-C: Representative Western blots showing protein expression of phospho-EGFR **A**: Y845 and **B**: Y1068 and EGFR and **C**: phospho-ERK1/2 and ERK in HL-1 cardiomyocytes after treatments with erlotinib or PP2. * $p \leq 0.05$, 1-way ANOVA with Holm-Sidak correction, N=5 independent experiments. **D**: Quantification of colocalization of N-CAD and DSG2 (Progen #610121 antibody) in HL-1 cardiomyocytes (Figure 1E), * $p \leq 0.05$, 1-way ANOVA with Holm-Sidak correction. Each data point represents one area, N=5 independent experiments. **E**: Maximum projections of immunostainings in HL-1 cardiomyocytes for N-CAD and DSG2 (ThermoFisher #PA5-79171 antibody) showing

an increase of DSG2 at the cell borders after erlotinib or PP2 treatments. Z-scans spanning the whole cell volume were performed using 0.25µm z-steps. White arrows indicate areas of increased DSG2 localization at the cell membrane. Scale bar: 10 µm. **F**: Quantification of colocalization of N-CAD and DSG2 (ThermoFisher #PA5-79171 antibody) in HL-1 cardiomyocytes, * $p \leq 0.05$, 1-way ANOVA with Holm-Sidak correction. Each data point represents one area, N=4 independent experiments.

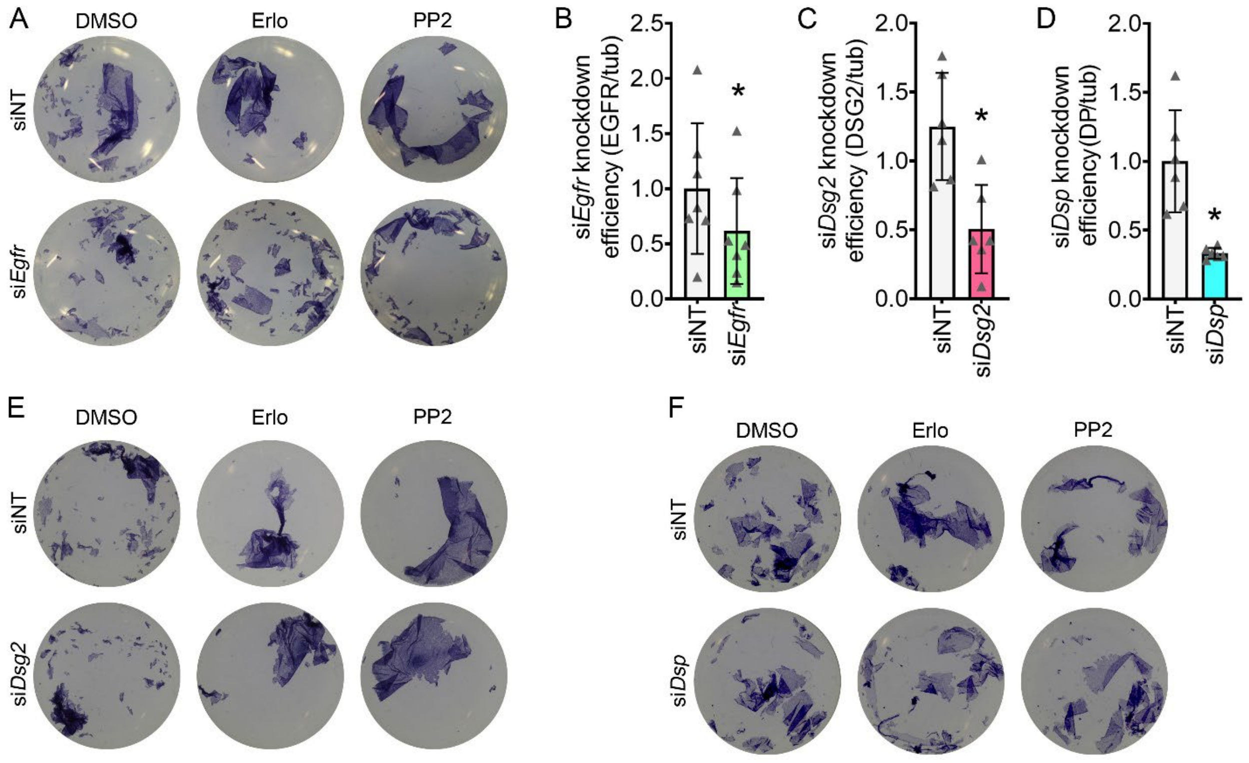

Figure S3 – Positive adhesiotropy induced by EGFR or SRC inhibition is dependent on DP

A:Representative pictures of the wells of the dispase-based dissociation assay in HL-1 cardiomyocytes after siRNA-mediated knockdown of *Egfr* (Figure 3A-B), **B-D**: Quantification of knockdown efficiency of **B**: *Egfr* **C**: *Dsg2* and **D**: *Dsp*. * $p \leq 0.05$, paired Student's *t*-test, N=6 independent experiments. **E**: Representative pictures of the wells of the dispase-based dissociation assay in HL-1 cardiomyocytes after siRNA-mediated knockdown of *Dsg2* (Figure 3E). **F**: Representative pictures of the wells of the dispase-based dissociation assay in HL-1 cardiomyocytes after siRNA-mediated knockdown of *Dsp* (Figure 3E).

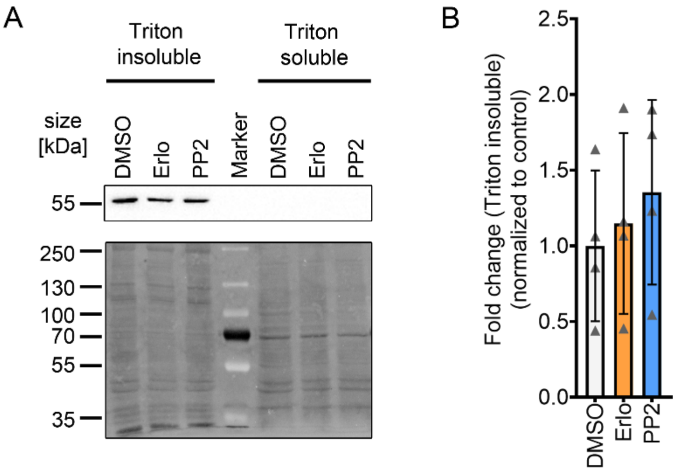

Figure S4 –DES protein levels upon EGFR or SRC inhibition

A: Representative Triton assay blots showing protein expression of DES. NoStain™ Protein Labelling Reagent served as loading control. **B**: Quantifications of Triton assay blots for DES. Only insoluble fraction was quantified, since no protein was detected in the Triton soluble fraction. 1-way ANOVA with Holm-Sidak correction, N=4 independent experiments.

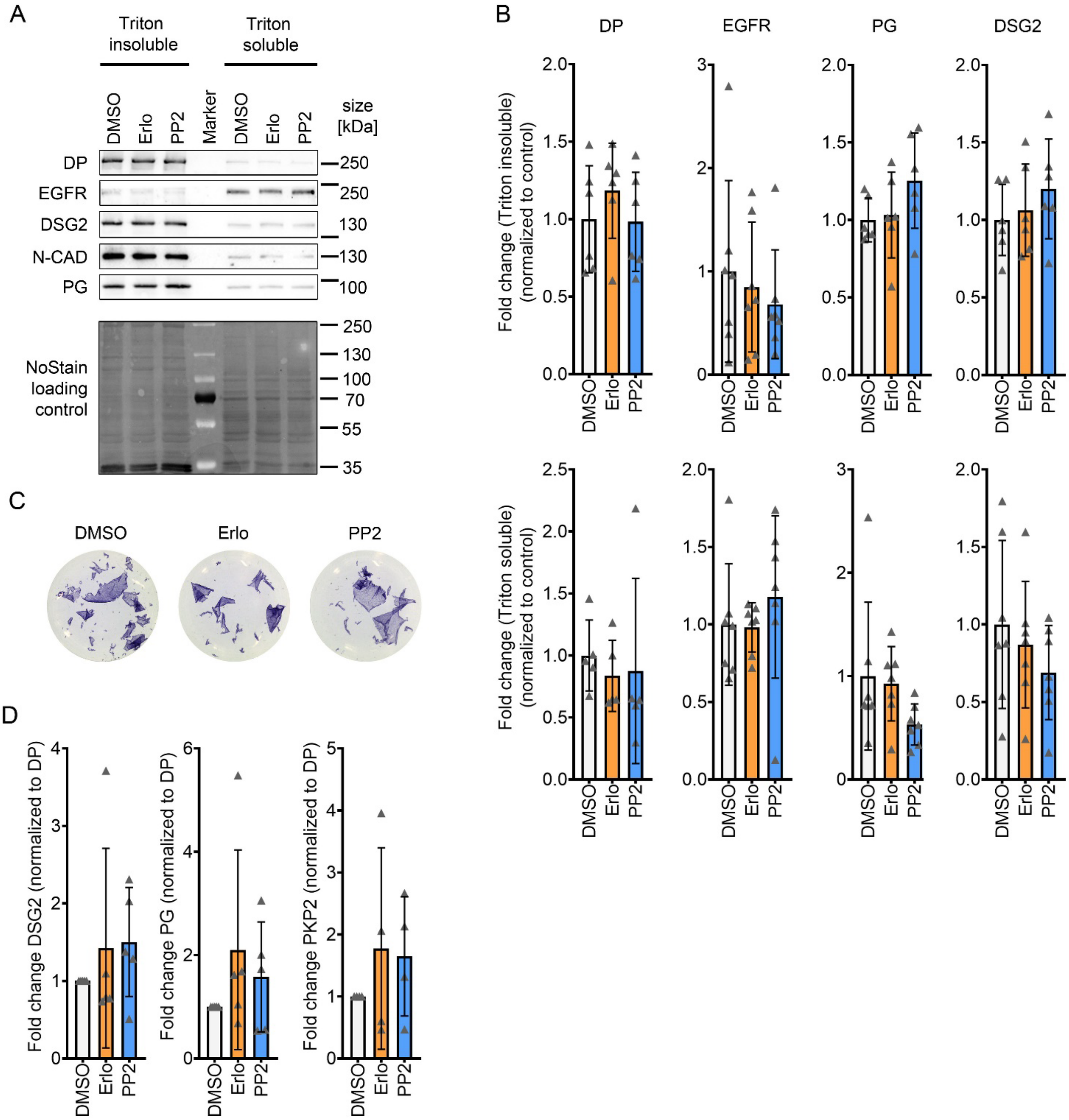

Figure S5 – EGFR or SRC inhibition do not cause changes in protein localization between detergent soluble and insoluble fractions

A: Representative Triton assay blots for DP, EGFR, PG and DSG2, NoStain™ Protein Labelling Reagent served as a loading control. Triton soluble fraction represents cytosolic proteins and membrane-bound proteins in Triton insoluble fraction. **B**: Quantifications of Triton assay blots for DP, EGFR, PG and DSG2 * $p \leq 0.05$, 1-way ANOVA with Holm-Sidak correction, N=5-7 independent experiments. **C**: Representative pictures of the wells of the dispase-based dissociation assay in HL-1 cardiomyocytes after 90 minutes of Ca^{2+}-depletion and subsequent

Ca^{2+}-repletion together with erlotinib PP2. **D**: Quantification of co-immunoprecipitated proteins DSG2, PG and PKP2 in DP immunoprecipitation (Figure 7G). Due to different pulldown efficiency of immunoprecipitation, samples were normalized to the respective DMSO control, 1-way ANOVA with Holm-Sidak correction, N=4-5 independent experiments.

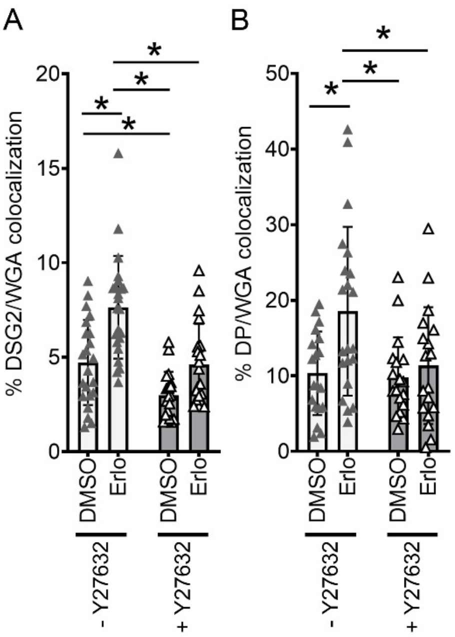

Figure S6 – Enhanced DSG2 and DP localization at the membrane is abolished upon inhibition of ROCK

Quantification of colocalization of **A**: DSG2 and WGA or **B**: DP and WGA (Figure 8E), * $p \leq 0.05$, 2-Way ANOVA with Holm-Sidak's multiple comparison test, N=5 independent experiments. Each data point represents one area.

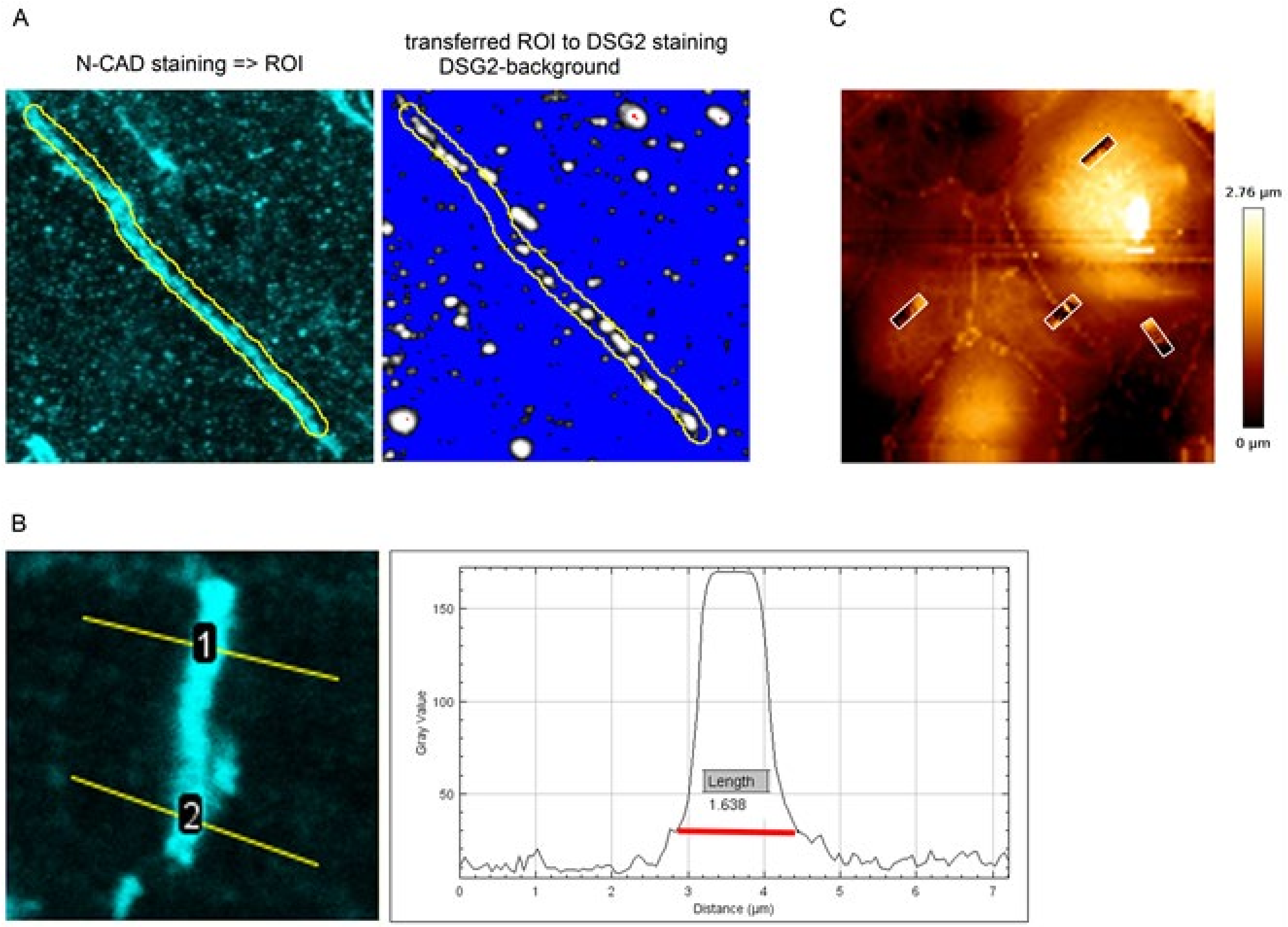

Figure S7 – Methods and quantifications explained

A: Quantification method for colocalization in immunostainings. The region of interest was chosen using N-CAD, and transferred to the image of the DSG2 staining. In this region, the amount of stained pixels was measured and a ratio DSG2 to N-CAD at the membrane was calculated. **B**: Quantification method for DP or DSG2 immunostaining width in murine cardiac slices. The width of respective protein staining was measured in two areas per ICD, the average values were calculated. WGA staining was used in order to orient the ICDs. Staining width was measured in areas, where WGA, DP and DSG2 staining was present. The measurements were performed using ImageJ as shown. **C**: Representative QI topography image of HL-1 cells with chosen areas for AFM measurements at cell borders and cell surfaces.

Table S1

PamGene Kinase assay after 15, 30 and 60 minutes of erlotinib treatment. Samples were compared to respective control-treated specimen. N=3 independent experiments. Kinase activity was considered to be altered, when the mean specificity score was above 1 and the significance score above 0.5
